# Biomaterial regulation of excessive inflammation restores osteoimmune homeostasis in diabetic bone regeneration

**DOI:** 10.1093/rb/rbag055

**Published:** 2026-03-10

**Authors:** Qiyue Zhou, Yu Zhang, Zhuo Dai, Qiang Li, Weijun Xiu, Haoxin Lv, Yongbin Mou, Heng Dong

**Affiliations:** Nanjing Stomatological Hospital, Affiliated Hospital of Medical School, Institute of Stomatology, Nanjing University, Nanjing, Jiangsu 210008, China; Nanjing Stomatological Hospital, Affiliated Hospital of Medical School, Institute of Stomatology, Nanjing University, Nanjing, Jiangsu 210008, China; Nanjing Stomatological Hospital, Affiliated Hospital of Medical School, Institute of Stomatology, Nanjing University, Nanjing, Jiangsu 210008, China; Nanjing Stomatological Hospital, Affiliated Hospital of Medical School, Institute of Stomatology, Nanjing University, Nanjing, Jiangsu 210008, China; Institute for Health Innovation and Technology, Biomedical Engineering Department, National University of Singapore, Singapore 119276, Singapore; Department of Oral Implantology, Suzhou doctor dental clinic Co. LTD, Suzhou 215000, China; Nanjing Stomatological Hospital, Affiliated Hospital of Medical School, Institute of Stomatology, Nanjing University, Nanjing, Jiangsu 210008, China; Nanjing Stomatological Hospital, Affiliated Hospital of Medical School, Institute of Stomatology, Nanjing University, Nanjing, Jiangsu 210008, China

**Keywords:** diabetic bone regeneration, osteoimmune homeostasis, immune dysregulation, bone tissue engineering

## Abstract

Diabetes mellitus markedly increases the incidence of fractures, implant failure and nonunion, primarily because chronic low-grade inflammation and a disrupted bone microenvironment impair regeneration. Under physiological conditions, coordinated interactions among immune, stromal, vascular and neural cells ensure timely initiation and resolution of inflammation, thereby maintaining osteoimmune homeostasis and supporting bone repair. In diabetes, hyperglycemia-induced oxidative stress, advanced glycation end products, impaired vascularization and ‘inflammatory memory’ prolong and intensify inflammatory responses. This excessive and unresolved inflammation disturbs immune–bone crosstalk, alters macrophage and T-cell phenotypes and uncouples osteogenesis and angiogenesis, ultimately hindering bone regeneration. This review summarizes the cellular and molecular basis of osteoimmune homeostasis and outlines how diabetes disrupts this regulatory network at systemic and local levels. We further highlight biomaterial strategies designed to modulate excessive inflammation and restore osteoimmune balance in diabetic bone regeneration, including localized delivery systems, cell-derived and extracellular vesicle-based agents, nanozyme-mediated microenvironmental regulation and immune-instructive physicochemical biomaterials. Finally, we discuss the critical hurdles of clinical translation (e.g. standardized scalable fabrication and long-term biosafety) and highlight multifactor integrated, logic-gated designs and systemic diabetes management strategies for advancing next-generation biomaterials for diabetic bone regeneration.

## Graphical abstract

**Graphical Abstract:**
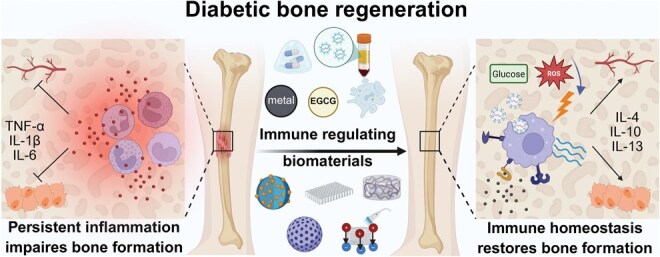

## Introduction

The global prevalence of diabetes has increased rapidly, leading to a growing burden of diabetes-related complications [1]. Clinically, diabetic patients present major challenges in both orthopedic and dental fields, particularly in procedures involving bone healing, guided bone regeneration and bone augmentation [2–4]. Although the etiologies of different types of diabetes vary, their shared pathological features, including hyperglycemia, chronic systemic inflammation and microvascular dysfunction, significantly increase the risk of delayed or impaired bone healing [5, 6].

Emerging evidence indicates that diabetes impairs bone regeneration not only through metabolic disturbances but also via immune dysregulation and sustained inflammation [7, 8]. Both systemic inflammatory activation and local immune imbalance induced by the hyperglycemic microenvironment disrupt normal bone repair [9, 10]. Additionally, impaired angiogenesis-induced hypoxia and paracrine dysfunction of mesenchymal stem cells (MSCs) and sensory nerve cells further sustain excessive inflammatory responses, ultimately impairing osteogenesis and vascularization [11, 12]. Glycemic control is considered beneficial for preventing skeletal complications and improving healing outcomes [13]. However, recent studies have revealed that glycemic fluctuations and prolonged hyperglycemia induce persistent epigenetic alterations and inflammatory memory in immune cells, limiting the effectiveness of glycemic control alone in restoring bone repair capacity [14, 15]. Some antidiabetic drugs and physical therapies show limited potential in enhancing bone regeneration, but clinical validation remains insufficient [16].

Biomaterials and tissue-engineering strategies offer promising alternatives for promoting bone repair [17–20]. However, biomaterials specifically designed to address the unique pathological microenvironment of diabetic bone healing are still under development. Given the preserved regenerative potential of bone tissue, advanced strategies should not only compensate for diabetes-induced impairments, but also actively support endogenous healing processes. A deeper understanding of diabetes-related disruptions, particularly immune dysregulation, is, therefore, critical for developing effective and personalized regenerative approaches [21, 22]. Consequently, biomaterial-based immunomodulatory interventions aiming to reconstruct the osteoimmune microenvironment have attracted increasing attention and demonstrated encouraging outcomes in preclinical studies.

**Figure 1:**
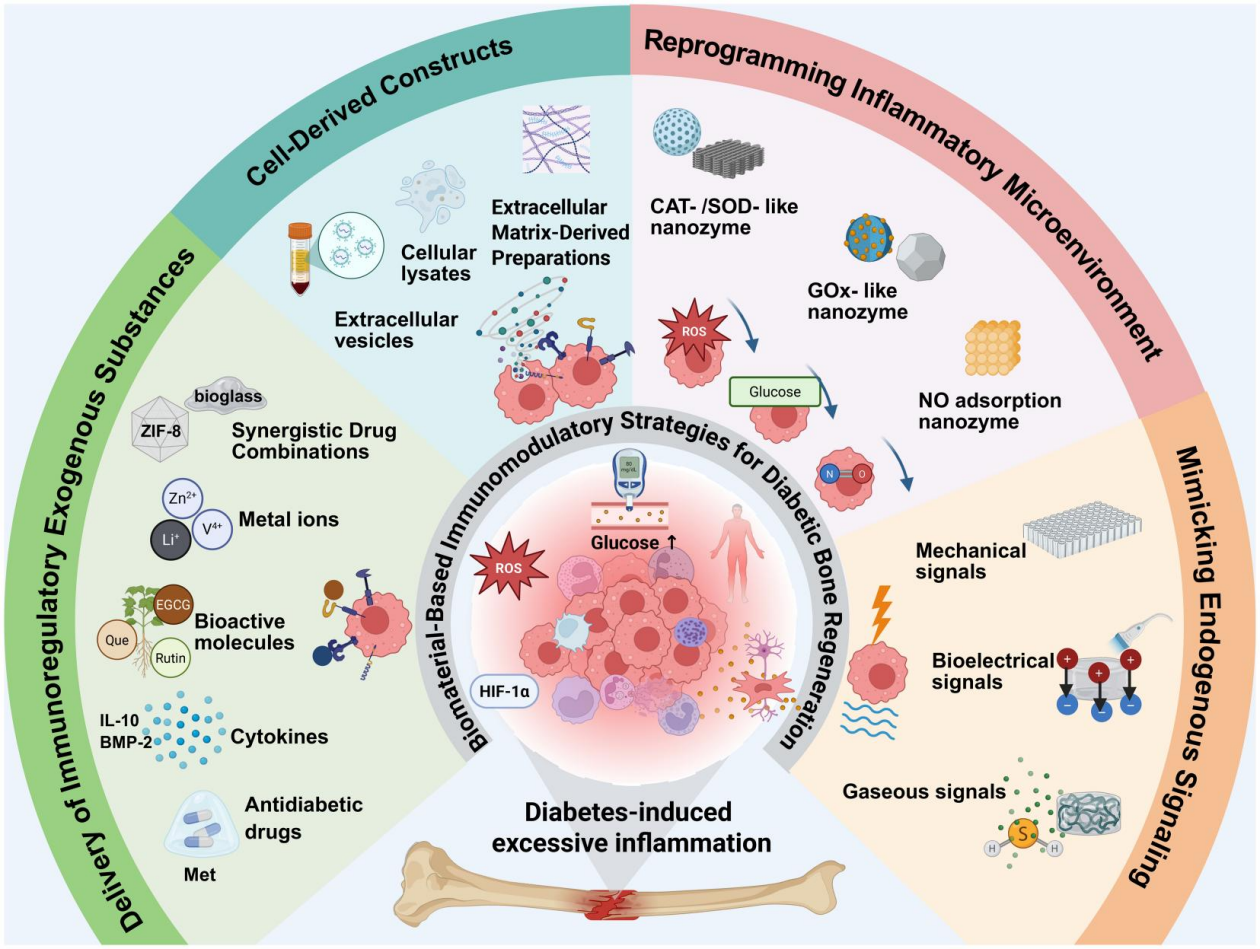
Schematic illustration of immune-modulating biomaterials for diabetic bone regeneration. Biomaterial strategies for modulating the dysregulated immune microenvironment in diabetes include: biomaterials designed for localized delivery of immunoregulatory agents, constructs derived from cells and extracellular vesicles, nanozyme-based systems that reprogram the inflammatory microenvironment, and immune-instructive physiochemical materials capable of providing precisely controlled spatiotemporal cues to restore osteoimmune homeostasis and promote bone regeneration.

Given these complex and interwoven pathological alterations, there is a pressing need to systematically integrate current mechanistic insights with emerging therapeutic approaches. This review aims to comprehensively summarize how immune dysregulation, which is driven by hyperglycemia, oxidative stress, aberrant paracrine signaling and impaired vascular-neural regulation, disturbs osteoimmune homeostasis and disrupts the sequential processes of inflammation resolution, osteogenesis and angiogenesis in diabetic conditions. Building upon this mechanistic foundation, we further present recent advances in the design of biomaterials capable of modulating excessive inflammation, rebalancing the diabetic bone microenvironment and promoting endogenous regenerative activity (Figure 1). These include biomaterial platforms for localized delivery of immunoregulatory agents, cell-derived and extracellular vesicle-based constructs, nanozyme-mediated microenvironmental reprogramming and immune-instructive physicochemical systems engineered to provide spatiotemporally controlled cues. Finally, we outline the remaining challenges in preclinical evaluation, safety assessment and translation to clinical application and offer future perspectives for developing next-generation osteoimmune-targeted biomaterials tailored for diabetic bone regeneration.

## Disruption of the physiological healing sequence in diabetic bone regeneration

**Figure 2:**
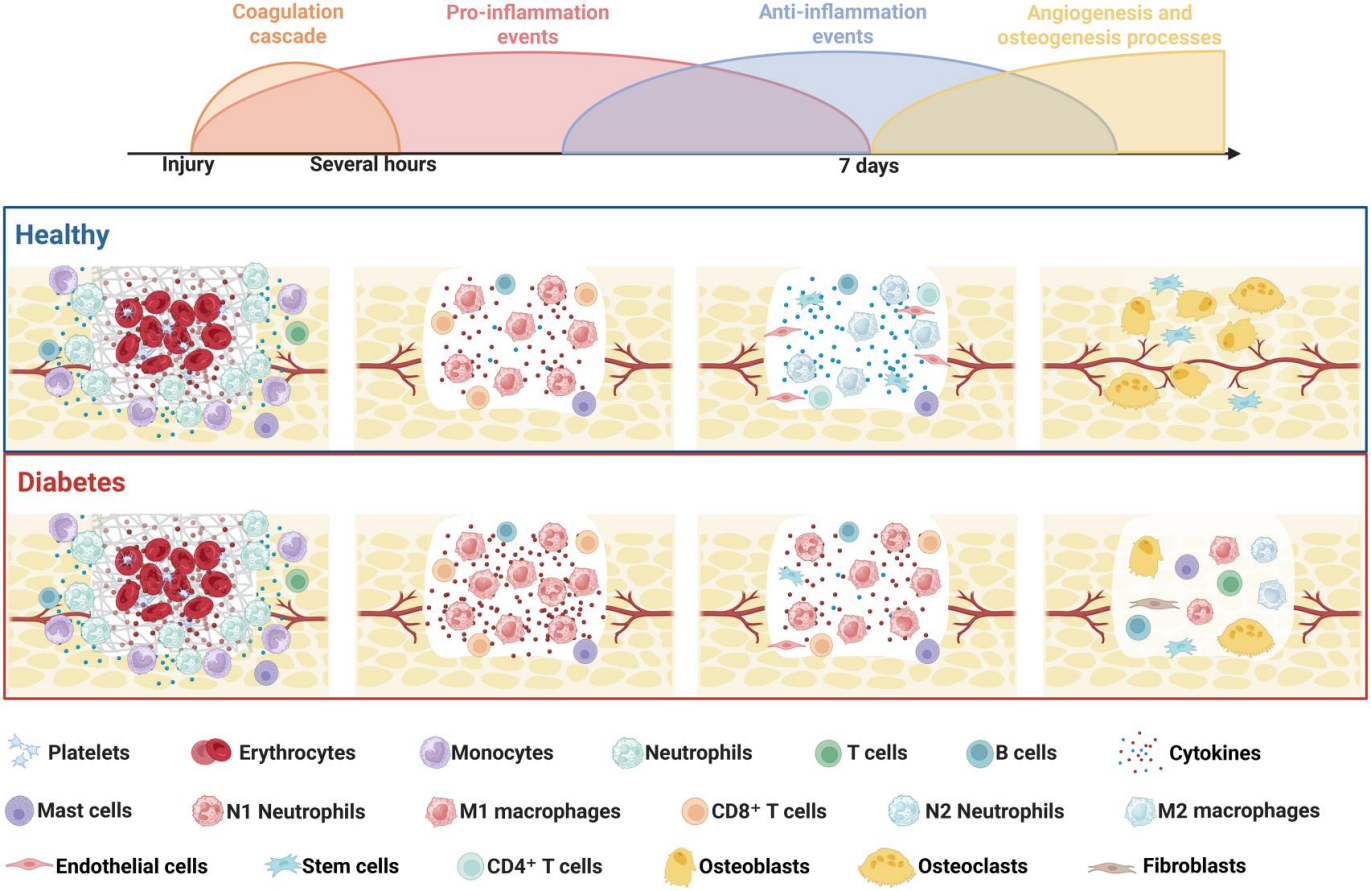
Schematic representation of the temporal progression of bone healing under healthy and diabetic conditions. The illustration of the coordinated phases of physiological bone repair, beginning with the coagulation Cascade immediately after injury, followed by acute pro-inflammatory events within several hours, a transition to anti-inflammatory and pro-resolving responses, and ultimately angiogenesis-coupled osteogenesis over the course of the first week. The spatiotemporal dynamics of cellular infiltration and immune modulation in healthy bone healing, characterized by timely neutrophil clearance, a balanced shift from M1 to M2 macrophages, effective recruitment of MSCs and well-coupled vascular and osteogenic regeneration. In contrast, the diabetic bone healing, where hyperglycemia-associated oxidative stress, persistent inflammatory cues, impaired angiogenesis and dysregulated immune cell phenotypes lead to prolonged neutrophil and M1 macrophage accumulation, insufficient M2 polarization, disrupted cytokine gradients and impaired osteoblast and endothelial cell recruitment. These alterations collectively delay inflammation resolution and compromise subsequent osteogenesis, ultimately contributing to impaired bone regeneration in diabetes.

As illustrated in Figure 2, physiological bone healing progresses through a tightly coordinated sequence of inflammatory, pro-resolving and regenerative events. Following injury, rapid activation of the coagulation cascade and recruitment of neutrophils and monocytes generate an acute but transient inflammatory phase. Timely clearance of neutrophils, macrophage polarization toward M2 phenotypes and the emergence of anti-inflammatory mediators create a permissive microenvironment for MSCs recruitment, angiogenic sprouting and subsequent osteogenesis. In diabetic conditions, this sequence is markedly disrupted. Hyperglycemia-induced oxidative stress, persistent inflammatory signaling and impaired angiogenesis prolong neutrophil and M1 macrophage accumulation, weaken M2 polarization and disturb cytokine gradients. These alterations delay inflammation resolution and impair the coupling between vascularization and osteogenesis, ultimately leading to compromised bone regeneration in diabetes.

## Osteoimmune homeostasis in physiological bone regeneration

Bone regeneration is a dynamic and tightly regulated process that proceeds through three overlapping phases: the inflammatory phase, the repair phase and the remodeling phase. Among these, the inflammatory phase serves as the critical initiating event that dictates the trajectory of subsequent healing. Immediately following injury, activation of the coagulation cascade and rapid recruitment of innate immune cells, particularly neutrophils and monocyte-derived macrophages, establish a transient pro-inflammatory microenvironment essential for debris clearance, pathogen defense and the release of early cytokine and chemokine signals.

The concept of osteoimmune homeostasis reflects the intricate bidirectional crosstalk between immune cells and bone-resident cells that governs this transition. A controlled and self-limiting inflammatory response, followed by timely resolution, is indispensable for successful bone repair. Central to this resolution process is the phenotype transition of macrophages from a pro-inflammatory (M1-like) to a pro-regenerative (M2-like) state, accompanied by the accumulation of regulatory immune cells and the secretion of anti-inflammatory mediators. This immune reprogramming restores local tissue homeostasis, promotes MSCs recruitment and osteogenic differentiation and facilitates angiogenic sprouting, which are fundamental to the repair phase. Although recent studies have demonstrated that macrophage phenotypes *in vivo* exhibit a wide range of different immunophenotypes with overlapping functional properties that extend beyond a simple binary classification, the M1/M2 concept has been widely adopted in immunology and biomaterials as a useful framework for understanding macrophage responses to diverse stimuli [23]. Therefore, throughout this review, we employ the M1/M2 framework to describe macrophage functional states, while acknowledging the inherent complexity and plasticity of macrophage polarization.

When osteoimmune regulation is balanced, these processes synergistically coordinate the formation of new bone matrix and its subsequent remodeling. Conversely, deviations in the magnitude or duration of inflammation disrupt immune-bone communication, impair MSCs function and angiogenesis and ultimately compromise the regenerative outcome. Thus, maintaining osteoimmune homeostasis is a prerequisite for effective physiological bone regeneration.

### Inflammatory phase

The healing process initiates immediately after injury with the formation of a hematoma, in which the fibrin-rich clot provides a provisional extracellular matrix that supports cellular adhesion, mechanotransduction and the spatial organization of early repair events [24]. Within this microenvironment, the innate immune system mounts a rapid pro-inflammatory response that defines the onset of the inflammatory phase. Platelet degranulation releases chemokines and inflammatory cytokines, such as monocyte chemoattractant protein-1 (MCP-1), which rapidly recruit neutrophils to the hematoma within hours, followed by monocyte infiltration and the establishment of an inflammatory cascade [25]. Neutrophils adopt a pro-inflammatory N1 phenotype, whereas infiltrating monocytes differentiate into classically activated M1 macrophages [25, 26]. These innate immune cells actively clear debris and pathogens and secrete essential pro-inflammatory mediators, including tumor necrosis factor-alpha (TNF-α), interleukin-6 (IL-6) and interleukin-1 (IL-1), that further amplify local inflammation and recruit additional immune and reparative cells [27, 28].

Although traditionally considered secondary responders, adaptive immune cells also contribute to the early inflammatory phase. T and B lymphocytes have been detected within the fracture hematoma during this stage [29, 30]. Among them, T cells play particularly prominent roles. Upon sensing injury-associated signals, T cells differentiate into cytotoxic CD8^+^ T cells that secrete interferon-gamma (IFN-γ) and TNF-α, thereby sustaining the early pro-inflammatory milieu [31, 32]. Crosstalk between T cells and macrophages, mediated by chemokines such as C-X-C motif chemokine ligands 9 and 10 (CXCL9/10) and cytokines such as interleukin-12 (IL-12), further amplifies and prolongs inflammatory signaling [33]. In addition, mast cells accumulate at fracture sites and regulate vascular permeability and cytokine release, influencing healing outcomes, particularly in severe fractures [32, 34].

A well-orchestrated early inflammatory response is now widely recognized as indispensable for successful fracture healing. Evidence from murine models demonstrates that local administration of low-dose recombinant human TNF-α (rhTNF-α) within the first 24 h post-injury enhances bone healing at 28 days [35]. This early TNF-α surge enhances neutrophil–monocyte/macrophage signaling and optimizes the progression to subsequent reparative phases. Conversely, excessive suppression of early inflammation, such as through systemic anti-TNF treatment or local delivery of interleukin-10 (IL-10), severely compromises fracture healing [35–38]. These findings underscore the importance of maintaining an appropriate magnitude and timing of inflammation to initiate downstream regenerative processes.

### Repair phase

Although the initial pro-inflammatory milieu is essential for debris clearance and immune activation, it is not conducive to osteogenesis, and therefore, must be rapidly resolved to permit tissue repair [25, 39]. Anti-inflammatory and pro-resolving signals emerge shortly after the establishment of the pro-inflammatory environment, typically within 24–36 h post-injury, and the transition from a predominantly pro-inflammatory state to a pro-regenerative one is generally completed by Day 7 [40].

This resolution of inflammation is mediated by a coordinated network of immune cells and anti-inflammatory cytokines, involving phenotypic transitions of neutrophils, macrophages and T lymphocytes [25]. As inflammatory stimuli diminish, neutrophils undergo apoptosis and are cleared via macrophage-mediated efferocytosis, an event that triggers the secretion of anti-inflammatory mediators such as interleukin-4 (IL-4), IL-10 and interleukin-13 (IL-13) and promotes macrophage polarization toward an M2 phenotype [41]. M2 macrophages play a pivotal role in orchestrating the repair phase, as murine studies demonstrate that the shift from M1 to M2 macrophages predominance temporally precedes the transition from inflammation to tissue repair [42]. Although emerging evidence highlights a spectrum of macrophage states and multiple M2 subsets *in vivo*, this review adopts the classical M1/M2 framework to emphasize their functional distinction in inflammation versus repair [43]. Meanwhile, newly infiltrated neutrophils can polarize into an anti-inflammatory N2 phenotype under microenvironmental cues [44]. The proportion of regulatory T cells (Tregs) and T helper 2 (Th2) cells also progressively increases during this stage, supporting inflammation resolution and promoting bone regeneration [26, 31, 45].

Timely resolution of inflammation is a prerequisite for initiating angiogenesis and osteogenesis. Hypoxia is a characteristic feature of the early bone-healing microenvironment [46], arising from vascular disruption and elevated oxygen consumption by infiltrating immune cells. Hypoxic tension activates adaptive signaling pathways, most notably the hypoxia-inducible factor (HIF) pathway, which induces transcription of hypoxia-responsive genes such as vascular endothelial growth factor (VEGF) and thereby promotes angiogenesis [47]. The establishment of new vasculature is essential not only for nutrient and oxygen delivery but also for supporting regenerative immune cell phenotypes, which rely primarily on oxidative phosphorylation rather than glycolysis to sustain their reparative functions [28].

### Remodeling phase

Following the resolution of acute inflammation, a pro-regenerative immune microenvironment is established, providing the necessary conditions for subsequent tissue repair [25, 38]. Newly formed blood vessels progressively alleviate local hypoxia, restore nutrient exchange and enable the recruitment of reparative stromal and progenitor cells, making vascular regeneration a fundamental prerequisite for osteogenesis [48]. In response to chemotactic cues such as transforming growth factor-beta (TGF-β), insulin-like growth factor-1 (IGF-1) and stromal cell-derived factor-1 (SDF-1) released within the healing niche, skeletal stem and progenitor cells migrate toward the injury site and commit to osteoblastic and osteoclastic lineages, initiating the deposition of woven bone [49].

Depending on anatomical context and mechanical conditions, bone formation proceeds through two major ossification pathways (endochondral and intramembranous ossification), both of which generate an initial network of mechanically weak woven bone [50]. The subsequent remodeling phase replaces this immature matrix with highly organized lamellar bone through a tightly coordinated process of osteoclast-mediated bone resorption and osteoblast-driven neo-ossification, ultimately restoring bone architecture and mechanical competence. During this stage, a second wave of T and B cell infiltration emerges following revascularization of the cartilage callus, and these lymphocytes actively contribute to the regulation of bone resorption and formation, thereby influencing the quality of remodeling [29].

Despite substantial progress, current findings reveal that inflammatory initiation, resolution and remodeling constitute an intricately coordinated cascade involving diverse immune and stromal cell populations and numerous molecular mediators. Many aspects of this regulatory network remain incompletely understood. For example, low-abundance immune cell subsets, including dendritic cells (DCs), basophils and natural killer (NK) cells, exhibit unique but poorly defined roles in bone metabolism [37, 51, 52]. The importance of neutrophils has also been revisited, with recent studies highlighting their functions in stem cell recruitment and the neutrophil-osteogenic cell axis [44, 53]. As osteoimmunology continues to evolve, the field is progressively uncovering the complex intercellular signaling networks that orchestrate bone regeneration. Cutting-edge technologies such as single-cell RNA sequencing, spatial transcriptomics and high-dimensional immune profiling now provide unprecedented resolution for investigating the immunological mechanisms that govern the remodeling phase [54–58].

## Diabetic disruption of osteoimmune homeostasis and excessive inflammation

Although the early phases of bone healing, including the initiation of inflammation and its timely resolution, are essential for successful regeneration, pathological conditions such as diabetes significantly disrupt these tightly regulated events. Numerous studies have demonstrated that the inflammatory phase in diabetic bone healing is both exaggerated and prolonged, failing to transition into an anti-inflammatory and pro-resolving microenvironment [59–62]. This impaired transition prevents the initiation of angiogenic and osteogenic programs, ultimately leading to delayed fracture healing and inferior bone regeneration outcomes. Consistent with this, experimental interventions that suppress persistent inflammation and promote the shift toward an anti-inflammatory milieu, such as the local delivery of IL-10, pro-resolving mediators or other immunoregulatory factors, have been shown to markedly enhance bone repair in type 2 diabetes mellitus (T2DM) models [60, 63].

**Figure 3:**
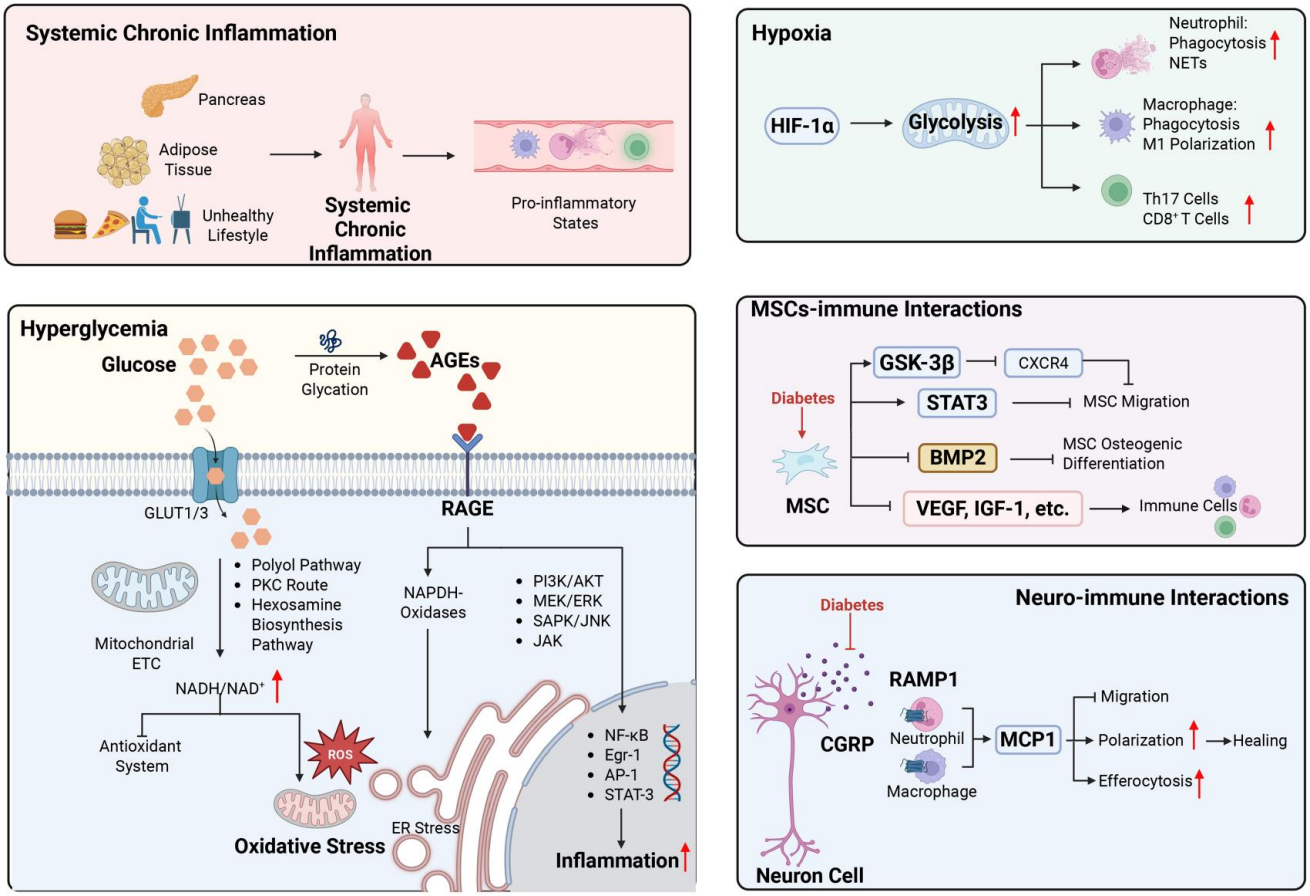
Schematic diagram of the major mechanisms driving diabetes-induced excessive inflammation. Diabetes promotes systemic chronic inflammation and hyperglycemia-induced metabolic stress, leading to mitochondrial dysfunction, AGEs-RAGE activation and excessive ROS production. Local hypoxia further enhances HIF-1α-dependent pro-inflammatory responses, including neutrophil activation, NETs formation and macrophage M1 polarization. Diabetes also impairs MSCs-immune crosstalk and disrupts neuro-immune signaling, collectively altering immune cell recruitment, polarization and efferocytosis. These systemic and microenvironmental abnormalities synergistically sustain unresolved inflammation in diabetic bone tissue.

The failure of inflammation resolution in diabetes arises from multifactorial mechanisms, involving both systemic and local disturbances. Systemically, diabetes is characterized by chronic low-grade inflammation driven by metabolic dysregulation, adipose tissue dysfunction and impaired endocrine signaling. Locally, hyperglycemia induces immune cell dysfunction through mechanisms such as reactive oxygen species (ROS) overproduction, advanced glycation end products (AGEs)–receptor for advanced glycation end products (RAGE) activation, mitochondrial stress and impaired antioxidant defense systems. These alterations collectively skew neutrophils, macrophages and T cells toward persistent pro-inflammatory phenotypes, disrupting osteoimmune balance. Moreover, a sustained pro-inflammatory microenvironment delays angiogenesis and osteogenesis, which further exacerbates immune dysregulation by reinforcing local hypoxia and altering MSCs and neuronal paracrine signaling pathways (Figure 3). Together, these factors create a self-perpetuating cycle of excessive inflammation and impaired regeneration that characterizes diabetic bone healing.

### Diabetes-associated systemic chronic inflammation

Patients with diabetes mellitus exhibit elevated levels of pro-inflammatory circulatory mediators [64]. Systemic chronic inflammation serves as both a triggering factor and a significant pathological hallmark of diabetes mellitus. Type 1 diabetes mellitus (T1DM) arises from a complex interplay of genetic susceptibility, environmental triggers and dysregulated immune responses, ultimately culminating in targeted autoimmune attacks against pancreatic β-cells. The resulting systemic inflammation is a consequence of both hyperglycemia and immunological aberrations [9, 65–67]. In T2DM, abnormal carbohydrate metabolism and the endocrine dysfunction of adipose tissue lead to a characteristic chronic low-grade inflammation. This inflammatory state triggers insulin resistance and progressively impairs pancreatic β-cells, ultimately culminating in the clinical onset of T2DM [68–70]. Recent evidence reveals that T2DM-induced systemic immune dysregulation shares pathophysiological parallels with the chronic, low-grade inflammatory state characteristic of aging [71]. Notably, during the pathogenesis of T2DM, senescent cells accumulate in adipose tissue, and their secretion of senescence-associated secretory phenotype (SASP) factors can promote insulin resistance in mouse models by interfering with insulin signaling, recruiting immune cells and other mechanisms. The pathophysiological processes of diabetes can also increase the accumulation of senescent cells and contribute to the development of various complications [69, 72]. Targeted disruption of the macrophage senescence cascade through rational biomaterial design ameliorates diabetic bone regeneration outcomes in rats [73].

Despite their distinct driving mechanisms, the induction of systemic low-grade inflammation resulting from multifactorial activation of innate immunity is a feature of both T1DM and T2DM. This systemic chronic inflammation renders circulating immune cells prone to exhibiting activated pro-inflammatory states. Periodontal macrophages in diabetic murine models exhibit nearly identical senescence-associated phenotypes to those in aged mice [72]. Neutrophils in diabetic patients demonstrate pathological hyperactivation marked by excessive superoxide production, elevated TNF-α secretion and dysregulated release of peptidyl arginine deiminase 4 (PAD4) and neutrophil extracellular traps (NETs) [74, 75]. T cells also undergo pro-inflammatory polarization characterized by diminished Th2 cell populations concomitant with expanded T helper 1 (Th1) and T helper 17 (Th17) subsets [76, 77].

### Alterations in hyperglycemic microenvironment

Hyperglycemia, a defining feature of diabetes mellitus, profoundly disrupts immune cell function through a series of intertwined metabolic and molecular mechanisms. Excessive extracellular glucose directly modulates immune cell phenotypes by inducing oxidative stress [10]. High glucose availability also promotes nonenzymatic protein glycation and the formation of AGEs, which structurally compromise extracellular matrix components and activate RAGE-mediated inflammatory signaling pathways, ultimately leading to cellular dysfunction [78]. Furthermore, emerging evidence indicates that hyperglycemia can induce ‘trained immunity’ within innate immune cells, whereby pro-inflammatory characteristics persist even after glycemic normalization [14, 79].

A central hallmark of the hyperglycemic microenvironment is oxidative stress imbalance, defined as a disruption between ROS production and the antioxidant defense system [80, 81]. Major ROS species, including superoxide (O_2_·^-^), hydrogen peroxide (H_2_O_2_), hydroxyl radicals (·OH) and singlet oxygen (^1^O_2_), are highly reactive [82, 83] and are primarily generated as metabolic by-products in the mitochondrial electron transport chain (ETC), as well as within the endoplasmic reticulum (ER) [84]. The antioxidant system consists of enzymatic antioxidants such as catalase (CAT) and superoxide dismutase (SOD), along with endogenous and dietary antioxidants [85]. In diabetic patients, ROS levels are markedly elevated [80]. Unlike many other cell types, immune cells do not rely on insulin-dependent glucose uptake; instead, they constitutively express glucose transporter 1 (GLUT1) and glucose transporter 3 (GLUT3) [86, 87]. Therefore, hyperglycemia leads to uncontrolled glucose influx, overwhelming mitochondrial metabolism and generating ROS levels exceeding the capacity of antioxidant defenses [85, 88].

In addition to mitochondrial overload, hyperglycemia causes extensive diversion of glycolytic intermediates into alternative metabolic pathways, further amplifying oxidative stress. Up to 30% of intracellular glucose is shunted into the polyol pathway, where aldose reductase converts glucose to sorbitol at the expense of nicotinamide adenine dinucleotide phosphate (NADPH) [89, 90]. Sorbitol is subsequently oxidized to fructose by sorbitol dehydrogenase, increasing the NADH/NAD^+^ ratio [91]. Meanwhile, the glycolytic intermediate diacylglycerol (DAG) activates the protein kinase C (PKC) pathway, which phosphorylates components of the NADPH oxidase complex and stimulates ROS production [92]. These mechanisms lead to elevated NADH/NAD^+^ ratios, NADPH depletion and ETC electron leakage, collectively producing excessive ROS [93]. NADPH depletion also impairs glutathione (GSH) regeneration, further weakening antioxidant capacity [94]. Additionally, fructose-6-phosphate enters the hexosamine biosynthesis pathway (HBP) to form uridine diphosphate N-acetylglucosamine (UDP-GlcNAc) [95], which contributes to ER stress through aberrant protein glycosylation [96].

Hyperglycemia also accelerates nonenzymatic protein glycation, a physiological process intensified under diabetic conditions [97]. Early glycation products such as glycated hemoglobin (HbA1c) and glycated albumin serve as biomarkers of glycemic status [98]. Prolonged hyperglycemia drives the formation of AGEs through irreversible molecular rearrangements [99]. Long-lived proteins such as collagen are particularly susceptible. AGE-mediated crosslinking (e.g. pentosidine) impairs mineralization, disrupts fibrillar alignment and weakens the biomechanical properties of bone, contributing to diabetes-associated bone fragility [100–106].

AGE accumulation initiates downstream pathology largely through the AGE–RAGE axis. RAGE is expressed on osteoblasts, osteoclasts, endothelial cells and multiple immune lineages including neutrophils, monocytes/macrophages, lymphocytes and DCs [96, 107]. RAGE activation has a profound impact on immune cells and is implicated in diabetes-related bone loss via its effects on monocyte-derived osteoclast precursors [108, 109]. Engagement of RAGE triggers multiple signaling pathways, including phosphatidylinositol 3-kinase (PI3K)/protein kinase B (PKB/AKT), mitogen-activated protein kinase kinase (MEK)/extracellular signal-regulated kinase (ERK), stress-activated protein kinase (SAPK)/c-Jun N-terminal kinase (JNK) and Janus kinase (JAK)/signal transducer and activator of transcription (STAT), leading to the upregulation of inflammatory and oxidative stress genes [110, 111]. In various diseases, the activation of RAGE on mononuclear phagocytes and lymphocytes triggers cellular activation and releases key proinflammatory mediators [96]. RAGE activation also promotes its own expression in a feed-forward amplification loop [112]. Moreover, AGEs and ROS reinforce one another bidirectionally: ROS accelerate AGE formation and AGE-RAGE signaling induces ROS via NADPH oxidase activation, forming a detrimental vicious cycle [96].

Recent studies also highlight that hyperglycemia induces epigenetically imprinted trained immunity, whereby innate immune cells adopt long-lasting pro-inflammatory phenotypes even after glucose normalization [14, 113]. The hyperglycemic milieu modulates a diverse spectrum of epigenetic modifications (encompassing DNA methylation, histone modification and noncoding RNA activity), thereby reshaping chromatin accessibility and orchestrating complex gene regulatory networks that remodel macrophage functional phenotypes during tissue repair [114]. This phenomenon is well documented in diabetic cardiovascular complications and likely influences diabetic bone regeneration, although further investigation is needed.

Therefore, excessive glucose exposure in the hyperglycemic microenvironment triggers mitochondrial overload, activation of multiple metabolic side pathways and impaired antioxidant systems, resulting in ROS overproduction and oxidative stress. Concurrently, AGE-RAGE signaling reinforces inflammatory phenotypes and oxidative damage. The persistence of hyperglycemia-induced epigenetic reprogramming ensures that these pro-inflammatory conditions continue even after glycemic control. Together, these mechanisms represent the core drivers of diabetes-induced immune dysregulation and contribute fundamentally to the chronic pro-inflammatory microenvironment that impairs bone regeneration.

### Sustained hypoxia

Chronic and unresolved hypoxia is a characteristic feature of diabetic wound and bone healing, largely resulting from impaired angiogenesis. This pathological hypoxic state arises from several convergent mechanisms. On one hand, the hyperglycemic and pro-inflammatory microenvironment directly damages vascular endothelial cells, compromising their proliferation, migration and tube-formation capacity [60, 115–120]. On the other hand, hyperglycemia suppresses both the stabilization and transcriptional activity of HIF-1, a master regulator of cellular adaptation to oxygen deprivation. Dysregulation of the HIF-1 pathway attenuates the hypoxic response and diminishes the expression of downstream angiogenic mediators, thereby impairing local neovascularization [11, 48].

As angiogenesis and osteogenesis are tightly coupled processes, insufficient vascular regeneration substantially hinders bone formation [121–123]. Newly formed blood vessels not only alleviate hypoxia and deliver essential nutrients but also guide the recruitment and differentiation of osteogenic progenitors. Therefore, sustained hypoxia contributes directly to deficiencies in osteoblast function and delayed bone regeneration.

Prolonged hypoxic tension also exacerbates immune dysregulation. Oxygen-sensitive pathways, including HIF-dependent metabolic reprogramming and nuclear factor kappa-B (NF-κB) signaling, drive immune cells toward increased aerobic glycolysis, promoting a shift to pro-inflammatory phenotypes and enhancing their chemotactic and cytokine-secreting activity [28, 124]. This metabolic polarization perpetuates inflammatory signaling, reinforcing a microenvironment that further inhibits angiogenesis. Thus, inflammation and impaired angiogenesis form a mutually reinforcing cycle in diabetic tissues.

Consistent with these mechanistic interconnections, therapeutic strategies that concurrently modulate inflammation and stimulate angiogenesis demonstrate superior efficacy. Compared with administering pro-angiogenic or anti-inflammatory agents alone, combined delivery significantly enhances vascular regeneration and promotes diabetic wound healing [125, 126]. These findings underscore the necessity of simultaneously addressing inflammatory and vascular defects to overcome the sustained hypoxia characteristic of diabetic bone regeneration.

### Paracrine regulation of other cells

Diabetes not only alters immune cell behavior directly but also disrupts the function of other key cellular components involved in bone repair. Systemic chronic inflammation and the hyperglycemic microenvironment impair the paracrine functions of MSCs, sensory neurons and other stromal cells, further aggravating immune dysregulation through multiple forms of intercellular crosstalk. Although the immunomodulatory roles of MSCs and the nervous system have only recently been recognized, accumulating evidence highlights their importance as regulatory nodes and potential therapeutic targets in diabetic bone regeneration.

During the reparative phase, MSCs serve as both osteogenic progenitors and potent immunomodulators. When exposed to inflammatory stimuli, MSCs secrete a wide range of immunoregulatory mediators [127]. In the presence of IFN-γ together with TNF-α or interleukin-1beta (IL-1β), MSCs become highly immunosuppressive, releasing factors such as indoleamine 2,3-dioxygenase (IDO), prostaglandin E_2_ (PGE_2_), nitric oxide (NO), TGF-β, hepatocyte growth factor (HGF) and heme oxygenase (HO) that inhibit T-cell proliferation, promote Treg differentiation and drive macrophage polarization toward an M2 phenotype [128–131]. By contrast, under low levels of pro-inflammatory cytokines, MSCs adopt a pro-inflammatory phenotype, producing chemokines such as macrophage inflammatory protein-1 (MIP-1), CXCL9 and CXCL10, which recruit lymphocytes and amplify early inflammatory responses [128]. Thus, MSCs exhibit substantial phenotypic plasticity, dynamically shaped by microenvironmental cues.

The diabetic milieu profoundly impairs MSCs function. Hyperglycemia and chronic inflammation suppress MSC migration by activating glycogen synthase kinase-3β (GSK-3β) to downregulate C-X-C chemokine receptor type 4 (CXCR4) expression and through IL-6/STAT3 signaling [132, 133], leading to reduced MSCs recruitment during bone healing [134]. MSCs from diabetic environments display diminished osteogenic differentiation and enhanced adipogenesis, partly due to reduced bone morphogenetic protein 2 (BMP-2) levels and impaired BMP signaling [135]. Their paracrine outputs are also altered, with reduced VEGF-A, lower IGF-1 expression and changes in exosomal cargo [136, 137]. These defects indicate that MSCs dysfunction contributes not only to poor osteogenesis but also to insufficient inflammation resolution, exacerbating healing impairment in diabetes.

The nervous system is another important regulator of bone regeneration. Sensory nerves respond to bone injury and influence regeneration through both direct neural signaling and paracrine mechanisms [138, 139]. Neural-derived factors regulate stem cell activity and modulate immune cell function, and this neuro-immune crosstalk is indispensable for mandibular bone regeneration in mice [140, 141]. Sensory neurons secrete calcitonin gene-related peptide (CGRP), which binds receptor activity-modifying protein-1 (RAMP1) on neutrophils and macrophages, inhibiting their recruitment, promoting apoptosis, enhancing efferocytosis and skewing macrophages toward a pro-repair phenotype [142, 143]. Loss-of-function studies, including CGRP antagonism and macrophage depletion, confirm that CGRP-macrophage signaling is a critical neuro-immune axis in bone regeneration [138]. Substance P (SP), another neuropeptide, exerts context-dependent effects: it can promote M1 polarization and increase IL-1β and TNF-α secretion [142], yet in laminectomy models, SP shifts macrophages toward an M2 phenotype via sphingolipid metabolism and induces NETs formation through C-X-C motif chemokine ligand 1 (CXCL1) [144].

Diabetic peripheral neuropathy, a common complication of diabetes, impairs neural structure and function, consequently interrupting neuro-immune interactions essential for tissue healing [12]. High-glucose-conditioned Schwann cell–derived exosomes have been shown to impair peri-implant bone formation when administered to healthy animals [145]. Conversely, exogenous CGRP delivery can partially restore impaired wound healing in diabetes by attenuating inflammatory activity [143]. Thus, diabetes-induced neural dysfunction contributes significantly to the pro-inflammatory microenvironment characteristic of impaired bone repair.

## Biomaterial-based immunomodulatory strategies for diabetic bone regeneration

Bone regeneration requires tightly coordinated interactions among immune, stromal, vascular and neural cells. In diabetes, persistent hyperglycemia, systemic chronic inflammation, sustained hypoxia and dysregulated paracrine signaling disrupt this coordination, preventing the timely transition from pro-inflammatory to pro-resolving immune phenotypes. These pathological obstacles can be summarized in three key aspects: (1) Elevated pro-inflammatory signaling; (2) Excessive ROS and AGEs; and (3) The compromised function of nonimmune regulators (such as angiogenesis, MSCs and sensory nerves) and their subsequent pro-inflammatory impact on the immune system during bone regeneration. To counteract these pathological barriers, recent smart biomaterial design strategies focus on reprogramming the diabetic osteoimmune microenvironment through three major strategies: (1) Targeted delivery of immunoregulatory or antidiabetic agents to restore inflammatory balance; (2) Removal or neutralization of pathological cues (such as ROS, excessive glucose or glycation intermediates); and (3) Stimulation of endogenous regenerative signaling via mechanical, bioelectrical or gaseous cues. These strategies aim to drive macrophages and other immune cells toward pro-regenerative phenotypes, restore immune-stromal communication and re-establish a permissive niche for angiogenesis and osteogenesis. The following sections summarize advances in immunomodulatory biomaterials, including localized drug delivery, cell-derived construct delivery, nanozyme-mediated microenvironmental reprogramming and immune-instructive physicochemical platforms capable of providing mechanical, bioelectrical or gaseous cues.

## Biomaterials for localized delivery of immunoregulatory exogenous substances

Targeted local delivery of immunoregulatory agents represents one of the most widely adopted biomaterial-based strategies for correcting diabetes-associated immune dysregulation. By providing high local concentrations of therapeutic substances while minimizing systemic exposure, these materials act directly within the impaired diabetic bone niche to guide immune cells toward pro-regenerative phenotypes. The exogenous agents incorporated into such systems include antidiabetic drugs that modulate metabolic inflammation, cytokines and chemokines that promote immune resolution, bioactive small molecules with anti-inflammatory or pro-angiogenic properties, and metal ions with demonstrated immunomodulatory functions (Table 1). Through controlled release, these biomaterial platforms aim to restore osteoimmune balance, facilitate macrophage polarization, enhance angiogenesis–osteogenesis coupling and ultimately improve bone regeneration in diabetic conditions.

**Table 1:** Targeted delivery of exogenous immunomodulatory substances for diabetic bone regeneration.

Delivery components	Biomaterials	Immune cell regulation mechanism	Ref.
Antidiabetic drugs	Injectable silk/sitagliptin gel scaffolds	Eliciting a stronger recruitment of M2 macrophages to the sites of Ti implants and a significant promotion of osteointegration	[146]
	Injectable ROS-triggered doxycycline and metformin delivery system	Suppressing inflammation and promoting bone regeneration	[147]
Cytokines	IL-10 loaded DNA hydrogel	Promoting M2 macrophage polarization to attenuate periodontal inflammation, triggering osteogenesis of MSCs	[148]
	IL-10 and BMP-2 loaded multistimuli-responsive hydrogel	Logic-based cargo release enables the regulation of macrophage polarization by remodeling the mitochondria-related antioxidative system, resulting in enhanced osteogenesis in diabetic bone defects	[149]
Bioactive molecules	EGCG loaded on controlled-release hydrogel	Ameliorating periodontal tissue inflammation and reducing the loss of alveolar bone through reducing inflammatory infiltration and collagen destruction	[150]
	EGCG loaded on dual stimulus responsive BSG scaffold	Directly regulating the shift of macrophages from M1 to the M2 phenotype by promoting autophagy and lessening the inhibition of autophagic flux, reducing the activation of NF-κB in stem cells and restoring its immunoregulatory capacity.	[151]
	EGCG loaded on ROS/glucose stimuli-responsive hydrogel	Favorable antioxidant and anti-inflammatory properties, along with a regulatory influence on the phenotypic transition of macrophages	[152]
	Que loaded on tetrahedral framework nucleic acids	Resisting oxidative stress and inflammatory responses	[153]
	Phlorotannin loaded on temperature-sensitive hydrogel	Attenuating inflammation levels, inhibiting osteoclast production, promote bone regeneration, inhibiting apoptosis and decreasing RAGE levels	[154]
	Rutin loaded on injectable bioresponsive bone adhesive hydrogel	Effectively mitigating oxidative stress, alleviating mitochondrial dysfunction and limiting the overactivation of NLRP3 inflammasome in macrophages	[155]
	Vitamin C loaded on self-adaptive bioactive scaffold	Antioxidative properties by scavenging the intracellular ROS, as well as promoting M2 polarization of macrophages	[156]
Metal ions	Lithium-modified bioglass-hydrogel	Relieving inflammation, providing an anti-inflammatory microenvironment for osteogenesis and angiogenesis	[157]
	Chondroitin sulfate lithium hydrogel	Regulating macrophage polarization to anti-inflammatory phenotype and alleviating the inhibition of angiogenesis and osteogenesis attributed to the activation of GSK-3β/β-catenin pathways	[158]
	ZIF-8 loaded PCL scaffold	Outstanding immunomodulatory and ROS scavenging capacities, which regulate M2 polarization of macrophages and drive functional cytokine secretion, thus, leading to a pro-regenerative microenvironment with enhanced vascularization	[159]
Drug synergism	Nanoparticles-Met@ZIF-8 modified hydrogel	Recovery of mitochondria and breakdown of the ROS-inflammation cascade cycle, thus, enhancing osteogenesis	[160]
	Met-loaded ZIF framework	Antiaging and immunomodulatory abilities by activating M2 macrophage polarization to secrete osteogenesis-related cytokines, also promoting bone regeneration	[161]
	PTHrP-1 anchored PDA-hybridized nanosized ZIF-8	Reducing ROS accumulation and maintain a favorable redox balance	[162]
	Que-encapsulated ZIF-8 nanoparticles	Inducing macrophage M2 polarization, scavenge excess ROS and restore mitochondrial membrane potential	[163]
	Bioactive Zn−V−Si−Ca glass nanoparticle hydrogel microneedles	Modulating the inflammatory microenvironment through downregulation of JAK-STAT and NF-κB inflammation signaling pathways	[164]
	Sr-MBGNs	Inhibiting the NOX-2/ROS/NLRP3/IL-1β axis	[165]

### Biomaterial-mediated local delivery of antidiabetic drugs

Although various oral antidiabetic drugs, including sitagliptin and metformin, have demonstrated beneficial effects on bone metabolism and repair, systemic administration alone is insufficient to fully rescue diabetes-impaired bone regeneration [2, 166]. Recent pharmacological studies have revealed that metformin, beyond its glucose-lowering function, directly targets mitochondria in both innate and adaptive immune cells, inhibits mitochondrial respiratory chain complex I, and regulates inflammatory responses through AMP-activated protein kinase (AMPK)-dependent and AMPK-independent mechanisms [167]. These immunometabolic properties provide a strong rationale for incorporating antidiabetic agents into biomaterials, enabling localized delivery to reprogram dysfunctional immune cells within diabetic bone defects.

**Figure 4:**
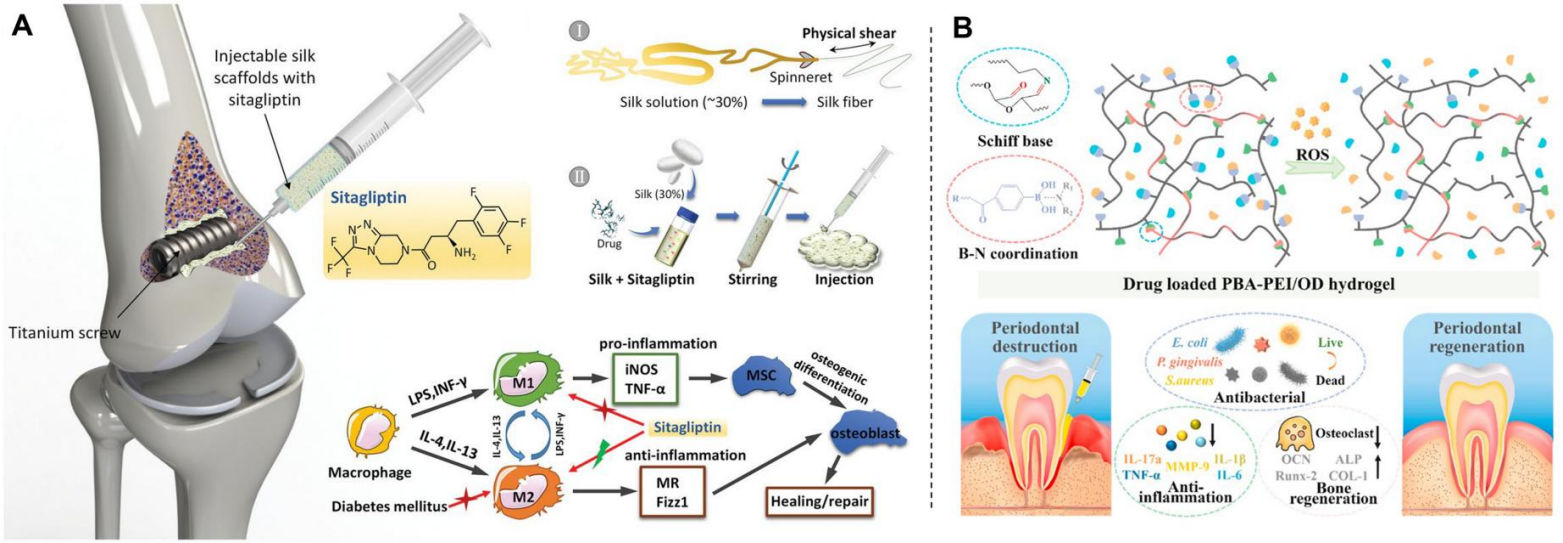
Antidiabetic drug delivery systems for diabetic bone repair. (**A**) Sitagliptin loaded in an injectable macroporous silk fibroin hydrogel enables localized enrichment of M2 macrophages at the titanium implant interface, thereby enhancing osseointegration under diabetic conditions. Reproduced with permission from Ref. [146]. Copyright 2020, Wiley. (**B**) Doxycycline and metformin co-delivered by a hydrogel composed of oxidized dextran and PBA-functionalized poly(ethylene imine) achieve synergistic anti-inflammatory effects (via metformin-mediated cytokine downregulation) and antibacterial activity (via doxycycline), addressing the limitations of monotherapy and enhancing the therapeutic potential for diabetic periodontitis. Reproduced with permission from Ref. [147]. Copyright2022, Elsevier.

In this context, several studies have explored biomaterial platforms as carriers for localized antidiabetic drug release. Xiang *et al.* developed an injectable macroporous silk fibroin hydrogel loaded with sitagliptin, demonstrating that local delivery (unlike oral administration) effectively enriched M2 macrophages at the titanium implant interface and significantly improved osseointegration (Figure 4A) [146]. Similarly, Zhao *et al.* [147] designed an injectable hydrogel composed of oxidized dextran and phenylboronic acid (PBA)-functionalized poly(ethylene imine), capable of co-delivering doxycycline and metformin. The dual-drug approach was motivated by prior observations in diabetic periodontitis, where monotherapy with either agent yielded limited therapeutic benefit (Figure 4B). In this composite system, metformin mainly exerted anti-inflammatory effects by reducing local cytokine expression, while doxycycline provided complementary antibacterial activity. Together, the synergistic actions of these agents enhanced local immune regulation and exhibited promising therapeutic potential for diabetic periodontitis.

### Biomaterial-mediated delivery of cytokines

Cytokines, as endogenous mediators of immune regulation, exert potent and highly specific effects on inflammatory resolution and tissue repair. Despite their therapeutic promise, their clinical translation is hindered by pharmacokinetic limitations, including rapid degradation, short half-life and difficulty in maintaining therapeutic concentrations at the injury site. Consequently, biomaterial-based delivery platforms have emerged as an effective strategy to achieve sustained, localized and bioactive cytokine release within the diabetic bone microenvironment.

**Figure 5:**
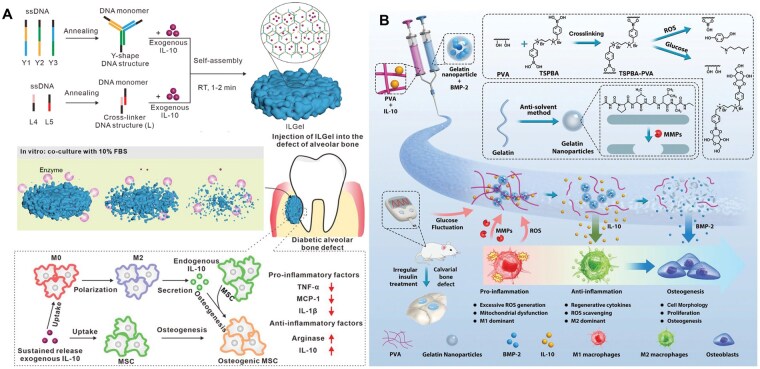
Cytokine-loaded biomaterials for diabetic bone defects. (**A**) Sustained release of IL-10 from a physically crosslinked DNA hydrogel significantly enhances alveolar bone defect healing in diabetic models. Reproduced with permission from Ref. [148]. Copyright 2022, American Chemical Society. (**B**) IL-10 and BMP-2 spatiotemporally sequentially released from a ‘diagnosis-treatment’ logic hydrogel in response to ROS and hyperglycemia reverse glucose fluctuation-induced inhibition of bone repair. Reproduced with permission from Ref. [149]. Copyright 2022, Wiley.

To address these limitations, various biomaterials have been engineered to stabilize cytokines and prolong their activity. For example, Li *et al.* [148] developed a physically crosslinked DNA hydrogel capable of sustained release of IL-10, a key anti-inflammatory cytokine essential for inflammation resolution and tissue regeneration. This hydrogel provided controlled IL-10 release for up to 7 days and significantly enhanced alveolar bone defect healing in diabetic models, achieving a 93.42 ± 4.6% defect healing rate at Day 21 compared with 63.30 ± 7.39% using free IL-10 (Figure 5A).

Given the temporally orchestrated nature of bone regeneration, achieving spatiotemporally programmed and sequential cytokine release, typically with early anti-inflammatory cues followed by later osteogenic signals, has become a major research focus. Li *et al.* developed a ‘diagnosis–treatment’ logic hydrogel that achieves temporally distinct release of IL-10 and BMP-2 [149]. The system integrates a reversible poly(vinyl alcohol) (PVA) network loaded with IL-10 and a colloidal gelatin nanoparticle network encapsulating BMP-2 (Figure 5B). In response to pathological stimuli such as ROS or hyperglycemia, the PVA network undergoes reversible cleavage, releasing IL-10 to mitigate excessive inflammation. Meanwhile, the slow degradation of gelatin nanoparticles allows BMP-2 release to peak after approximately 5 days, aligning with the onset of osteogenesis. This multistimuli-responsive, biocascade regulation strategy not only maximizes cytokine synergism but also adapts to inflammation exacerbated by glycemic fluctuations. *In vivo* results demonstrated complete rescue of glucose fluctuation-induced inhibition of bone repair, achieving osteogenesis comparable to healthy controls.

### Biomaterial-mediated delivery of bioactive molecules

**Figure 6:**
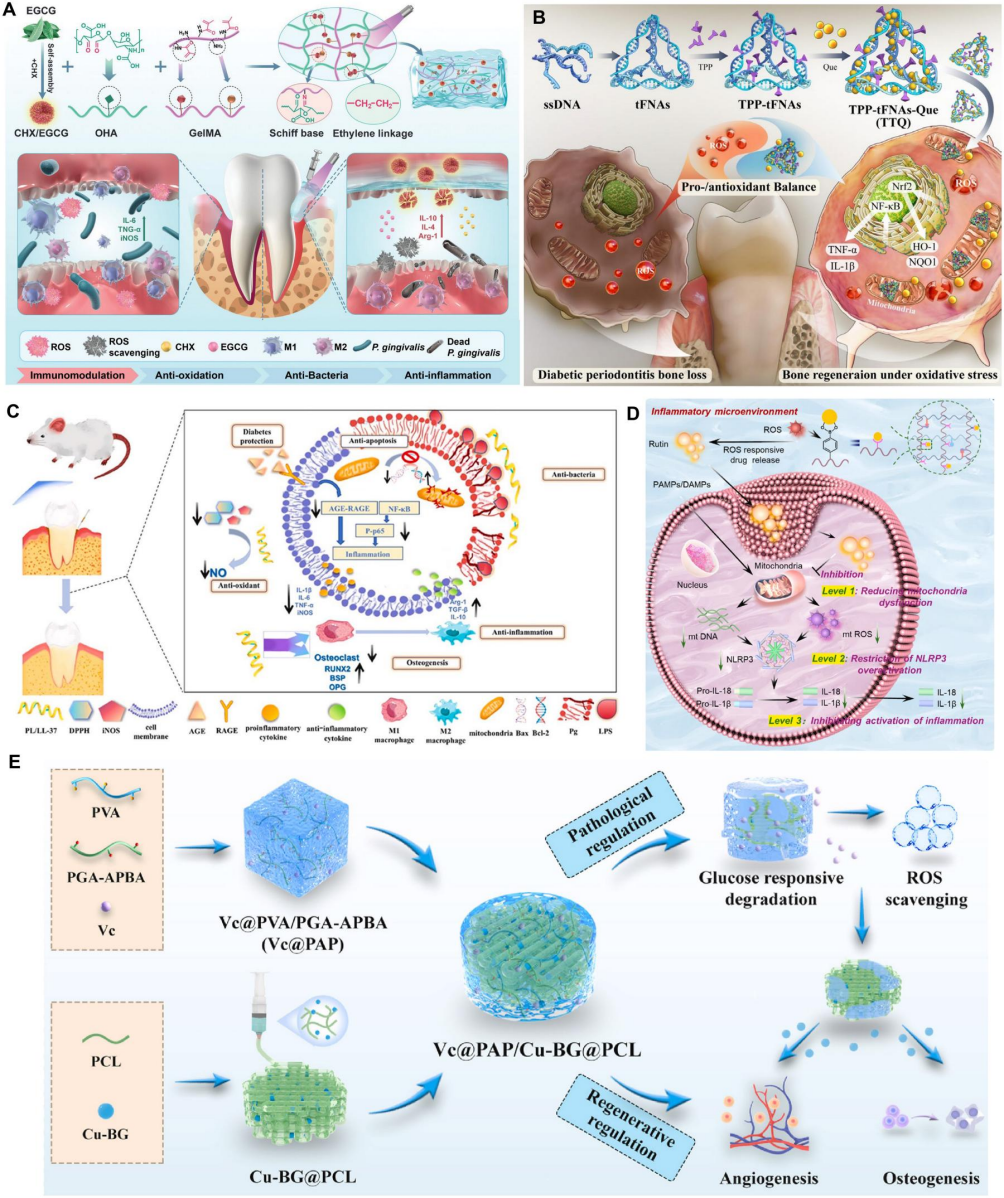
Bioactive molecules delievery systems for diabetic bone defects. (**A**) EGCG sustained-release systems can regulate macrophage polarization and promote diabetic bone repair. Reproduced with permission from Ref. [150]. Copyright 2025, Wiley. (**B**) Que encapsulated within tFNAs, a mitochondria-targeted delivery system which reduce oxidative stress and inflammation to promote diabetic periodontal repair. Reproduced with permission from Ref. [153]. Copyright 2024, Elsevier. (**C**) Seaweed-derived PL and antimicrobial peptide LL-37 co-encapsulated in a hydrogel exhibited antibacterial, anti-inflammatory and osteogenic activities, shifting the hyperglycemia-aggravated inflammatory periodontal microenvironment to one conducive to tissue repair. Reproduced with permission from Ref. [154]. Copyright 2025, Elsevier. (**D**) Rutin conjugated to a ROS-sensitive hydrogel scavenged mitochondrial ROS, inhibited NLRP3 inflammasome assembly in macrophages, and established an anti-inflammatory microenvironment favorable for bone formation. Reproduced with permission from Ref. [155]. Copyright 2025, Elsevier. (**E**) VC incorporated into a glucose-responsive scaffold attenuated local inflammation via its release and coordinated the release of Cu/Si ions to enhance angiogenesis and osteogenesis, improving diabetic bone repair. Reproduced with permission from Ref. [156]. Copyright 2024, Elsevier.

A variety of plant-derived and naturally occurring bioactive molecules have recently gained attention as promising immunomodulators for diabetic bone regeneration. Polyphenolic compounds, in particular, exhibit antioxidant, anti-inflammatory and immunoregulatory properties that can be harnessed through biomaterial-based delivery systems. For example, Ge *et al.* [150] selected epigallocatechin-3-gallate (EGCG), a green tea–derived polyphenol with broad biological activity. They engineered a controlled-release hydrogel composed of gelatin methacryloyl (GelMA) and oxidized hyaluronic acid, which sustained the local release of EGCG together with chlorhexidine acetate (Figure 6A). In diabetic periodontitis models, this system significantly reduced macrophage- and neutrophil-derived inflammatory cytokines and achieved therapeutic outcomes comparable to minocycline, the current clinical standard. Additionally, EGCG has been used in other biomaterial platforms to modulate macrophage phenotype and promote the repair of diabetic alveolar bone defects [151, 152].

Other natural compounds with documented immunomodulatory effects have also been employed. Li *et al*. [153] utilized quercetin (Que, a flavonoid with strong antioxidant and anti-inflammatory activity) as a therapeutic agent (Figure 6B). By encapsulating Que within tetrahedral framework nucleic acids (tFNAs), they created a mitochondria-targeted delivery system capable of reducing oxidative stress and inflammatory responses, ultimately promoting periodontal tissue repair in a diabetic model. Likewise, Li *et al.* [154] co-encapsulated phlorotannins (PL), a seaweed-derived antidiabetic polyphenol, with the antimicrobial peptide LL-37 in a hydrogel platform (Figure 6C). Their system exhibited a broad range of beneficial effects, including antibacterial, anti-inflammatory, antioxidant, osteogenic and anti-apoptotic activities, effectively shifting the periodontal microenvironment from a hyperglycemia-aggravated inflammatory state to one conducive to tissue repair.

Rutin, another natural flavonoid, represents a particularly promising candidate due to its ability to suppress protein domain-associated protein 3 (NLRP3) inflammasome activation [155]. When conjugated to hydrogels through a ROS-sensitive linker, rutin can be released in response to oxidative stress. The resulting system functions as a mitochondrial ROS scavenger, inhibits NLRP3 inflammasome assembly in macrophages and establishes an anti-inflammatory microenvironment favorable for bone formation (Figure 6D).

In addition to polyphenols, essential nutrients with antioxidant properties have also been incorporated into biomaterials. Ascorbic acid (vitamin C, VC), known for its capacity to directly neutralize free radicals and reduce intracellular ROS, has been used to mitigate inflammation within diabetic bone defects. Cai *et al*. [156] incorporated VC into a glucose-responsive scaffold based on PBA-grafted polyglutamic acid and polyvinyl alcohol. This system enabled VC release to attenuate local inflammation and subsequently coordinated the release of copper (Cu) and silicon (Si) ions to enhance angiogenesis and osteogenesis, ultimately improving diabetic bone repair (Figure 6E).

### Biomaterial-based delivery of metal ions

Metal ions actively participate in numerous immunoregulatory pathways, and their capacity to modulate intracellular signaling and immune cell behavior provides a strong mechanistic basis for developing metal-ion-based biomaterials for diabetic bone regeneration [168]. By locally releasing metal ions at controlled rates, these materials aim to reshape the diabetic inflammatory microenvironment, promote macrophage phenotype transition and enhance downstream osteogenic and angiogenic responses.

**Figure 7:**
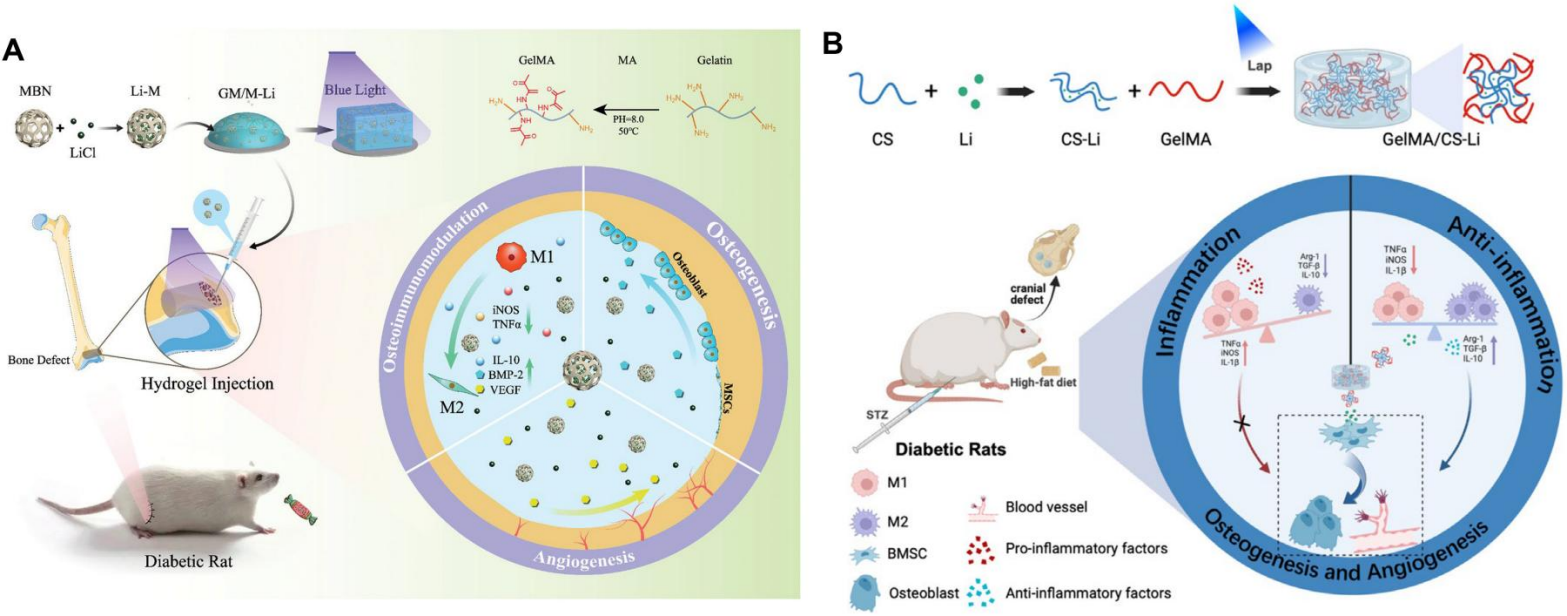
Metal ion-based biomaterials for diabetic bone regeneration. (**A**) Li-modified bioglass-hydrogel system reprogrammed macrophages, attenuated inflammation, stimulated osteogenesis/angiogenesis and in diabetic models via GSK-3 inhibition. Reproduced with permission from Ref. [157]. Copyright 2022, Wiley. (**B**) CS-Li compound enhanced GSK-3β inhibition and Wnt/β-catenin-mediated macrophage polarization via Li-CS coordination, improving *in vivo* osteogenesis under diabetic conditions. Reproduced with permission from Ref. [158]. Copyright 2025, Elsevier.

Lithium (Li^+^) is one of the most extensively studied immunomodulatory metal ions. Wu *et al.* [157] designed a Li-modified bioglass-hydrogel system capable of releasing Li^+^ to attenuate inflammation and stimulate osteogenesis and angiogenesis. Although complete repair of critical-sized bone defects was not achieved in diabetic models, the material effectively reprogrammed macrophages from pro-inflammatory toward pro-regenerative phenotypes (Figure 7A). The therapeutic effect is primarily attributed to Li-mediated inhibition of GSK-3, a kinase that is aberrantly activated in the diabetic microenvironment, thereby modulating downstream pathways to achieve anti-inflammatory and osteogenic outcomes [169]. To further improve the biological activity of Li^+^, Xu *et al.* [158] synthesized a chondroitin sulfate-lithium (CS-Li) compound (Figure 7B). The coordination of Li^+^ with CS enhanced the inhibition of GSK-3β activity and promoted Wnt/β-catenin pathway–mediated macrophage polarization, resulting in improved osteogenesis under diabetic conditions *in vivo*.

Zinc (Zn^2+^), widely recognized for its osteogenic capacity, has recently been shown to possess significant immunomodulatory effects as well [170]. Wu *et al.* [159] engineered a stimuli-responsive delivery platform based on zeolitic imidazolate framework-8 (ZIF-8), a representative metal-organic framework (MOF) capable of controlled Zn^2+^ release. In this system, periodic near-infrared (NIR) irradiation together with the acidic diabetic microenvironment serves as the trigger for Zn^2+^ release, enabling precise spatiotemporal control. Photothermal stimulation combined with Zn^2+^-mediated signaling enhanced macrophage infiltration and drove macrophage polarization toward the M2 phenotype. This immunoregulatory effect accelerated angiogenesis and mobilized endogenous stem cells during the early inflammatory phase, ultimately supporting timely transition into the proliferative and remodeling stages of tissue repair.

### Biomaterial-based delivery of synergistic drug combinations

**Figure 8:**
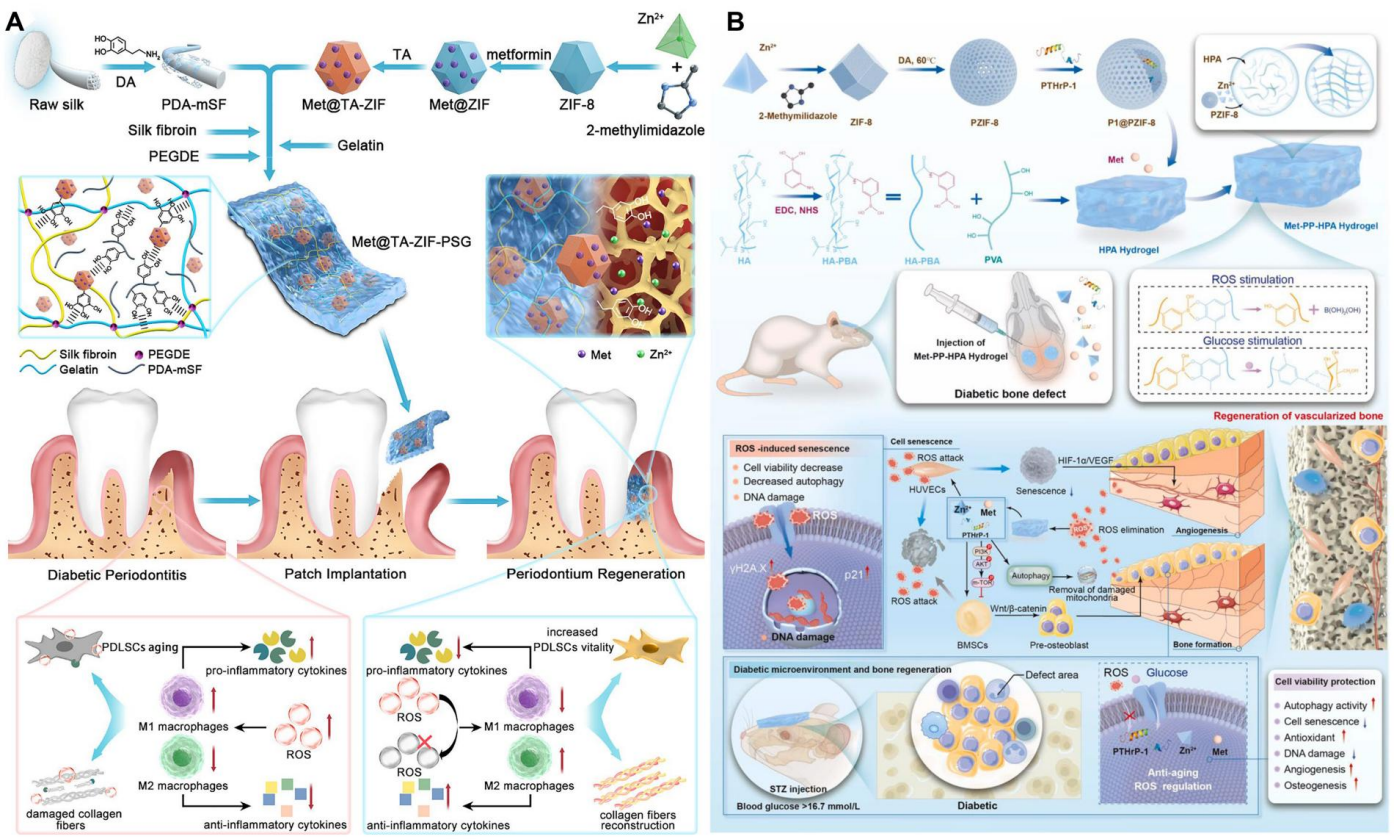
Synergistic drug delivery biomaterials for diabetic bone regeneration. (**A**) Metformin-loaded ZIF-8 incorporated into silk scaffold achieved complementary immunoregulation and ROS inhibition, enhancing diabetic bone repair *in vitro*/*in vivo*. Reproduced with permission from Ref. [163]. Copyright 2023, American Chemical Society. (**B**) PTHrP-1@ZIF-8 incorporated into a metformin-loaded hydrogel endows the system with enhanced osteogenic and angiogenic capabilities, building upon its inherent immunomodulatory function. Reproduced with permission from Ref. [162]. Copyright 2026, Elsevier.

To enhance therapeutic efficacy in the complex diabetic bone microenvironment, many studies have integrated multiple immunomodulatory agents into composite biomaterials, allowing synergistic interactions that surpass the effects of single-agent delivery. A representative example involves the incorporation of metformin into MOFs. Met@ZIF-8, a metformin-loaded zeolitic imidazolate framework, has been incorporated into various scaffold systems [160, 161]. This dual-delivery platform provides a coordinated release of Zn^2+^ and metformin, resulting in complementary mechanisms: Zn^2+^ supports immunoregulation and osteogenesis, while metformin attenuates ROS-driven inflammatory cascades and preserves mitochondrial homeostasis in immune cells (Figure 8A). Shi *et al*. [162] loaded parathyroid hormone-related peptide-1 (PTHrP-1) onto Met@ZIF-8. By virtue of the sustained and prolonged release of PTHrP-1, this composite system further promotes osteogenesis and angiogenesis. Together, these effects significantly enhance diabetic bone repair in both *in vitro* and *in vivo* models (Figure 8B).

Combined delivery systems of bioactive molecules and ZIF-8 have also achieved favorable osteogenic effects in diabetic models. Wang *et al*. [163] loaded Que-encapsulated ZIF-8 nanoparticles (Que@ZIF-8) into a GelMA biomimetic gel. By scavenging excessive ROS and restoring mitochondrial membrane potential, this system induces M2 polarization of macrophages, thereby remodeling the pathological microenvironment of diabetes to promote angiogenesis and osteogenesis.

Other studies have explored multi-ion or multi-molecule synergistic strategies based on bioactive glasses. Li *et al.* [164] developed hydrogel microneedles incorporating Zn-V-Si-Ca glass nanoparticles, in combination with gallic acid. The coordinated release of Zn^2+^, vanadium (V^4+^, V^5+^) and gallic acid modulated the immune microenvironment by downregulating the JAK-STAT and NF-κB inflammatory pathways. Another study constructed a 3D-printed strontium-containing mesoporous bioactive glass nanoparticle (Sr-MBGNs)/GelMA composite scaffold [165]. Through the synergistic release of strontium (Sr^2+^), calcium (Ca^2+^) and Si ions, the scaffold inhibits the NADPH oxidase 2 (NOX-2)/ROS/NLRP3/IL-1β axis, thereby achieving the conversion of the diabetic bone defect site to an anti-inflammatory microenvironment. This multicomponent approach demonstrated improved immunoregulatory effects and promoted tissue repair under diabetic conditions.

Although numerous immunomodulatory agents have demonstrated promising anti-inflammatory activity, single-agent delivery often fails to fully restore the impaired regenerative capacity associated with diabetes. The multifactorial nature of diabetic bone pathology typically requires simultaneous targeting of metabolic stress, oxidative injury, immune dysregulation and defective osteogenesis. Consequently, multidrug delivery strategies that integrate agents with distinct, complementary mechanisms are increasingly adopted.

## Cell-derived constructs and extracellular vesicles

The use of cell-derived constructs represents an evolution from traditional stem cell transplantation approaches. Rather than delivering living cells, this strategy administers bioactive cellular components that retain the ability to initiate and regulate tissue regeneration. By avoiding the transplantation of whole cells, cell-derivative-based therapies reduce concerns related to tumorigenicity, immunogenic rejection and low cell survival rates in the harsh diabetic microenvironment. These acellular constructs also offer practical advantages, including improved engineerability, higher structural stability, superior tissue penetration and greater compatibility with advanced biomaterial platforms [171].

Cell derivatives, such as cell lysates, extracellular matrices and extracellular vesicles (EVs), contain a rich repertoire of bioactive molecules, including metabolites, proteins, DNA, mRNA, miRNA and lipids. When locally delivered, these components can form a complex anti-inflammatory and pro-regenerative signaling network that counteracts the excessive inflammation characteristic of diabetes. Numerous studies have demonstrated the therapeutic potential of various cell derivatives in diabetic bone regeneration, with representative applications summarized in Table 2. For example, EVs released from MSCs carry cytokines, growth factors and regulatory RNAs that modulate macrophage polarization, restore impaired angiogenic signaling and promote osteogenic differentiation [177]. Through these multifactorial actions, cell-derived constructs provide a powerful and versatile approach for reprogramming the diabetic bone microenvironment and enhancing endogenous healing.

**Table 2:** Targeted delivery of active cellular components for diabetic bone regeneration.

Active cellular components	Biomaterials	Immune cell regulation mechanism	Ref.
Extracellular vesicles	M2 macrophage-derived exosomes	Inducing the conversion of M1 macrophages into M2 macrophages by stimulating the PI3K/AKT pathway	[63]
	M2 macrophage-derived extracellular vesicles decorated implants	Inhibiting NLRP3 inflammasome activation in M1 macrophage and reducing the levels of inflammatory cytokines such as IL-1β by targeting NEK7	[172]
	M2 macrophage-derived extracellular vesicles loaded DNA	Regulating macrophage polarization and promoting the expression of proliferative and osteogenic factors	[173]
	MSC-derived extracellular vesicles decorated implants	Impelling phenotypic alterations in macrophage polarization via multipathway regulation, decreasing proinflammatory M1 macrophage formation	[174]
Cellular lysates	Macrophage lysate loaded on hydrogel	Anti-inflammatory cytokine networks enhancing vascularized bone repair	[175]
Extracellular matrix preparations	Porcine SIS based hydrogel	Exerting immunomodulatory effects via the suppression of the NLRP3 signaling pathway and the elevation of adhesion-related protein expression	[176]

### Extracellular vesicles

EVs are 30 nm to 5 μm phospholipid bilayer-enclosed nanostructures that encapsulate a wide range of bioactive substances, including metabolites, proteins and nucleic acids (DNA and RNA) [178]. They are released by virtually all immune and tissue-resident cells and function as intercellular messengers by delivering their molecular cargo to recipient cells, thereby orchestrating complex signaling networks that regulate immune responses and cellular behavior [179]. EVs derived from various cell types exert significant roles in both physiological and pathological processes, including immunomodulation, angiogenesis and differentiation. For example, EVs from MSCs or M2 macrophages can remodel the immune microenvironment and enhance bone repair [180]; whereas EVs from bone marrow-derived MSCs (BMSCs) exposed to hyperglycemic conditions display impaired regenerative capacity, contributing to compromised bone defect repair [136]. This functional disparity underscores the paramount importance of the cellular source in the biomanufacturing of EV-based therapeutics, and highlights their potential as a precision strategy for restoring the dysregulated osteoimmune microenvironment in diabetic bone regeneration.

**Figure 9:**
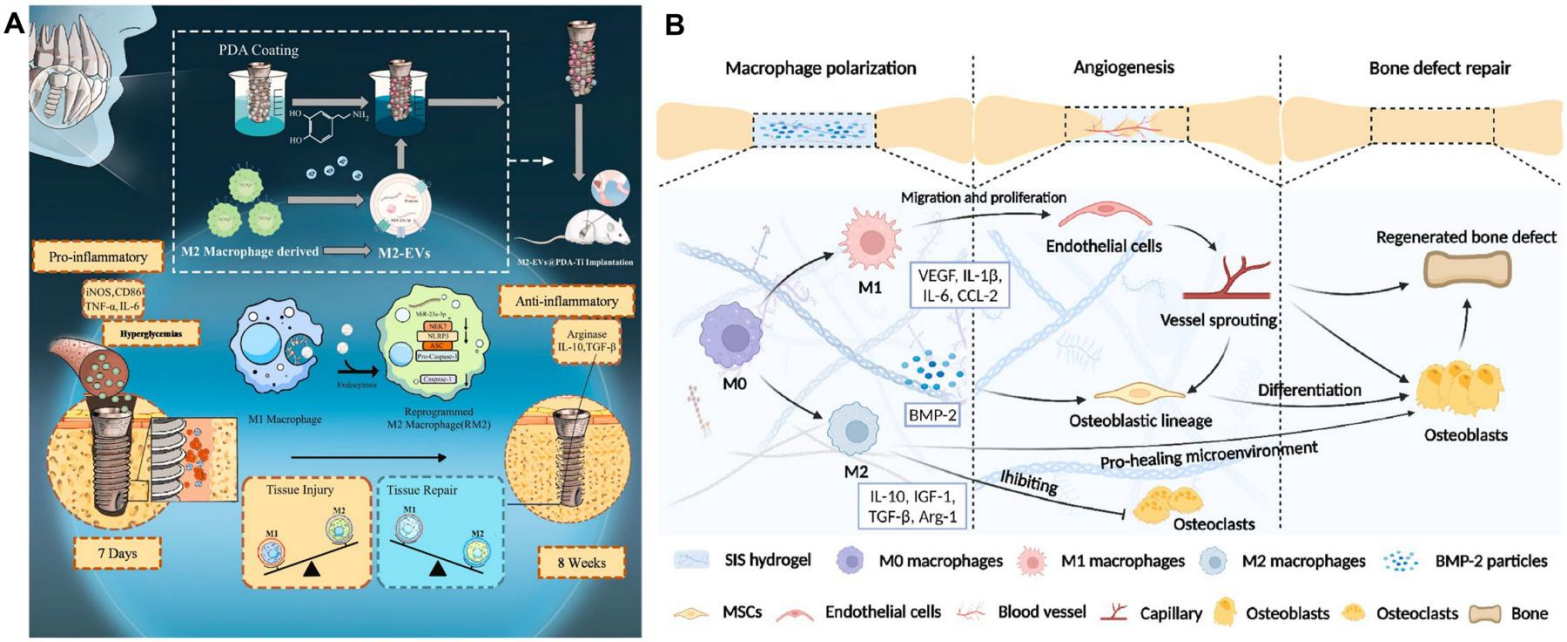
EV-based strategies for diabetic bone regeneration. (**A**) M2 macrophage-derived EVs immobilized on titanium implants enhanced osseointegration by delivering miR-23a-3p, which downregulated NEK7 expression and inhibited NLRP3 inflammasome activation, ultimately promoting M1-to-M2 macrophage repolarization. Reproduced with permission from Ref. [172]. Copyright 2025, Elsevier. (**B**) SIS hydrogel could sequentially induce the macrophages to polarize into M1 and M2 phenotypes, which later were revealed to be conducive to promoting angiogenesis. Reproduced with permission from Ref. [176]. Copyright 2022, Elsevier.

Several studies have demonstrated the immunoregulatory capacity of EVs in diabetic models. Wang *et al*. [63] extracted exosomes derived from M2 macrophages and applied them to fracture sites in diabetic mice, resulting in increased M2 macrophage accumulation and improved fracture healing. The therapeutic effect was mediated predominantly through activation of the PI3K/AKT pathway, which promoted macrophage reprogramming within the defect microenvironment. Similarly, M2 macrophage–derived EVs immobilized onto titanium implants via a polydopamine coating enhanced osseointegration by delivering miR-23a-3p, which downregulated NEK7 expression and inhibited NLRP3 inflammasome activation, ultimately promoting M1-to-M2 macrophage repolarization (Figure 9A) [172].

Despite their potent biological functions, free EVs exhibit rapid clearance and limited retention at the delivery site. To address this limitation, Peng *et al*. [173] developed a DNA hydrogel constructed from DNA-templated silver nanoclusters to encapsulate M2-EVs, achieving sustained release for up to 10 days. Another approach employed a biomimetic peptide containing lysine and noncoding levodopa (DOPA) functionalized with a dibenzocyclooctyne (DBCO) group [(DOPA)_6_-PEG_5_-DBCO] to immobilize MSC-derived EVs on titanium surfaces through biorthogonal click chemistry, allowing stable anchoring of EVs on the implant interface [174]. Although the long-term release kinetics of this interface remain under investigation, this strategy provides a promising route for EV tethering.

Beyond natural EVs, advances in bioengineering have enabled the development of engineered EVs with enhanced stability, targeting specificity and therapeutic potency [181]. While existing modification strategies for EVs have not further enhanced their immunomodulatory function, they have improved the capacity to promote angiogenesis and osteogenic differentiation. For instance, EVs loaded with zinc finger E-box binding homeobox 1 (ZEB1) have been shown to promote angiogenesis-dependent osteogenesis in diabetic conditions [182]. Additionally, melatonin-loaded M2 exosomes effectively induced immunometabolic reprogramming and improved bone repair in periodontitis-associated diabetic models [183].

EV-based biomaterials hold considerable translational promise due to their biocompatibility, low immunogenicity and capacity for complex biological regulation. Nevertheless, several challenges remain. EVs contain diverse proteins, lipids and nucleic acids and their precise mechanisms of action require further elucidation. Moreover, the therapeutic activity of natural EVs is influenced by the physiological state of the parent cells, and their inherent potency may be insufficient for severe diabetic conditions. Engineered EVs provide a more controllable and tunable alternative, while plant-derived EVs have recently emerged as additional candidates with anti-inflammatory properties and potential applicability in diabetic bone regeneration [184].

### Cellular lysates and extracellular matrix-derived preparations

Compared with extracellular vesicles, whole-cell lysates offer simpler preparation procedures and contain a broader spectrum of regulatory components, including cytokines, enzymes and metabolites. These features endow cell lysates with strong immunomodulatory potential. Yue *et al*. [175] reduced gluconeogenic flux in THP-1 macrophages by knocking down mitochondrial phosphoenolpyruvate carboxykinase 2 (PCK2), thereby promoting M2 macrophage polarization. Lysates derived from these reprogrammed macrophages were enriched with anti-inflammatory factors and were subsequently incorporated into a hydrogel system. When applied under diabetic conditions, the construct effectively reshaped the local immune microenvironment by suppressing excessive inflammation and facilitating tissue repair.

Extracellular matrix-derived biomaterials represent another promising class of cytokine-enriched constructs. These materials are produced through decellularization techniques that remove immunogenic cellular components while preserving native structural proteins, biochemical signals and matrix-bound cytokines. Owing to this biomimetic preservation, extracellular matrix-derived scaffolds closely replicate natural tissue microenvironments and are particularly well suited for bone regeneration. For example, small intestinal submucosa (SIS)-derived hydrogels retain cytokine networks capable of promoting macrophage polarization toward anti-inflammatory phenotypes (Figure 9B) [185]. However, given the complex pathological characteristics of diabetic bone defects, extracellular matrix preparations alone may be insufficient to fully restore immune homeostasis. Supplementation with growth factors such as BMP-2 or bone morphogenetic protein 4 (BMP-4) has been shown to enhance their immunomodulatory and osteoinductive efficacy [176, 185].

Although cellular lysates and extracellular matrix-derived scaffolds contain abundant anti-inflammatory components, their therapeutic effects rely primarily on local release into the surrounding microenvironment rather than the targeted transport achieved through EV-mediated intercellular communication. This difference in delivery mechanism may contribute to their relatively limited potency when compared with engineered EV-based systems. Further optimization of targeted delivery, controlled-release profiles and synergy with other biomaterials may enhance the translational potential of these acellular constructs for diabetic bone regeneration.

## Nanozyme-based systems for reprogramming the inflammatory microenvironment

As discussed above, excessive accumulation of glucose and ROS in diabetic bone defects drives persistent pro-inflammatory activation. This pathological state provides a strong rationale for the application of nanozymes (engineered nanomaterials with intrinsic enzyme-like catalytic properties) to eliminate ROS, glucose and other harmful metabolites and thereby restore immune homeostasis [186]. By mimicking natural enzymatic activities such as peroxidase, CAT, superoxide dismutase and oxidase reactions, nanozymes can efficiently modulate redox balance within the diabetic microenvironment (Table 3).

**Table 3:** Employment of nanozymes for diabetic bone regeneration.

Functional classification	Biomaterials	Immune cell regulation mechanism	Ref.
Enhanced ROS scavenging	Injectable magnesium-gallate bioenzyme-reinforced hydrogel	Facilitating the scavenging of overexpressed ROS, promoting macrophage polarization to the M2 phenotype, and attenuating early-stage inflammation	[187]
	Cerium-containing mesoporous bioactive glass nanoparticles	Scavenging diabetes-induced ROS, promoting macrophage polarization towards the anti-inflammatory phenotype, and accelerating early osseointegration	[188]
	CeO_2_ nanodots with Na_2_TiO_3_ nanotubes constructed on Ti implants	Catalyzing ROS thoroughly into benign O_2_ and H_2_O, relieving the oxidative stress of microenvironment and accelerating M2 macrophage phenotype polarization	[189]
	Cerium-tannic acid formed ‘chain armor’	Regulating HIF-1α activity by reducing the level of mitochondrial ROS, effectively alleviating mitochondrial dysfunction and reprogramming macrophages to a pro-healing state	[190]
	Carbon dots	Effectively scavenging superoxide anion and hydrogen peroxide, and promoting vascularization by activate PI3K-AKT signaling pathway	[191]
	Guanidination carbon dots decorated sulfonated polyether ether ketone	Promoting osteointegration by inhibiting the pro-inflammatory factors and enhancing the anti-inflammatory factors	[192]
	Ultrathin WOx nanoribbons	Remodeling metabolic patterns for achieving 86.3% M2 macrophage induction in a high glucose microenvironment	[193]
Reduced local glycosylation	Bimetallic MOF-derived Mn@Co_3_O_4_@Pt nanoenzyme	Disrupting the glucose-ROS-induced inflammation and promoting osteogenesis and angiogenesis	[194]
	Au@Pt nanoparticles loaded on hydrogel	Efficient glucose consumption and ROS elimination concurrently, promoting the osteogenic differentiation and paracrine capabilities of BMSCs, and subsequently inhibiting inflammation and enhancing angiogenesis	[195]
	Au@cyclodextrin/linear functionalized ferrocene@glucose oxidase	Effectively inhibiting the vicious cycle of ROS-inflammatory cascade through catalytic cascade reactions, up-regulating the expression of heat shock proteins under near-infrared irradiation, which promotes periodontal bone repair	[196]
	Glucose oxidase and catalase-assisted biomineralized calcium phosphate nanosheets	Relieving inflammation in diabetes by scavenging overproduced H_2_O_2_, stimulating neovascularization and facilitating new bone formation	[197]
NO regulator	Ruthenium nanozymes loaded on hydrogel	Orchestrating macrophage reprogramming by neutralizing ROS and reversing NO-mediated mitochondrial metabolism, rejuvenating MSCs and endothelial cells within diabetic mandibular defects, and producing newly formed bone with quality comparable to that of normal bone	[198]

Compared with natural enzymes, nanozymes offer several critical advantages, including enhanced physicochemical stability, resistance to degradation, adjustable catalytic efficiency and the ability to integrate multiple functions into a single platform [199]. These features make nanozymes particularly well suited for the complex and dynamic pathological environment of diabetic bone defects. Beyond rebalancing ROS and glucose levels, nanozyme-mediated microenvironmental regulation benefits multiple aspects of tissue repair, including promoting the transition of immune cells toward pro-regenerative phenotypes and supporting angiogenesis and osteogenesis. Together, these effects contribute to a more favorable microenvironment that facilitates coordinated bone regeneration.

### Nanozymes with enhanced ROS scavenging capacity

**Figure 10:**
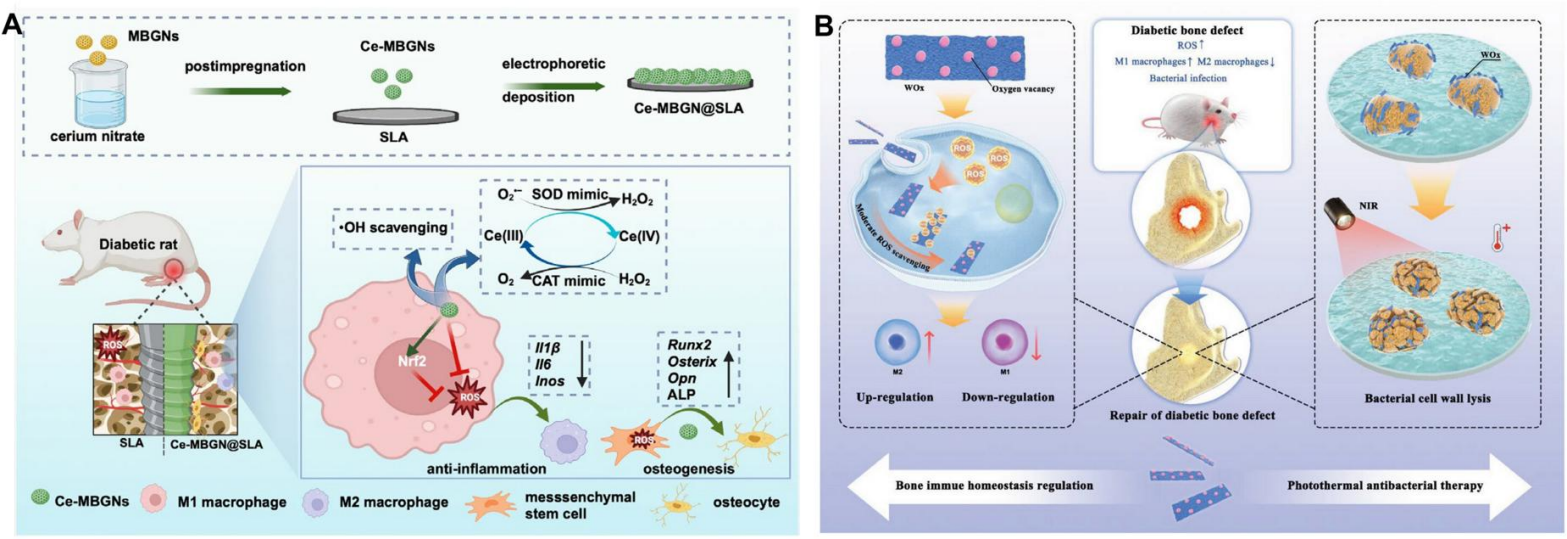
ROS-scavenging nanozymes for diabetic bone regeneration. (**A**) CeO_2_ nanozyme effectively scavenged ROS and promotes M2 macrophage polarization. Reproduced with permission from Ref. [188]. Copyright 2024, Springer Nature. (**B**) WO_x_ nanoribbons with moderate ROS-scavenging ability. Reproduced with permission from Ref. [193]. Copyright 2024, Wiley.

A wide range of engineered nanomaterials have been designed to mimic the catalytic functions of endogenous antioxidant enzymes such as SOD and CAT. These nanozymes enable efficient ROS detoxification within the diabetic microenvironment, thereby restoring redox homeostasis and supporting immune modulation. Duan *et al.* developed a magnesium-gallate (MgGA) bioenzyme with SOD- and CAT-like activities and incorporated it into a hydrogel scaffold, achieving potent ROS-scavenging effects and improved immune regulation [187]. Similarly, another research group combined cerium oxide (CeO_2_) nanozymes with mesoporous bioactive glass nanoparticles to fabricate a functional coating on titanium implant surfaces through electrophoretic deposition (Figure 10A) [188]. CeO_2_ nanodots immobilized on polydopamine coatings have also demonstrated strong SOD- and CAT-like activities [189]. In addition, cerium nanoparticles coordinated with tannic acid can self-assemble into a metal-phenolic network coating on orthopedic implants, forming a protective ‘chain armor’ structure that effectively scavenges ROS, promotes M2 macrophage polarization and enhances osseointegration in diabetic models [190].

Carbon dots represent another promising class of ROS-scavenging nanomaterials. As zero-dimensional carbon-based structures smaller than 10 nm, carbon dots exhibit excellent functionalizability and versatile physicochemical properties [200]. Zhang *et al.* [191] synthesized carbon dots capable of reducing intracellular ROS levels and restoring endothelial cell function. Building on this foundation, the team developed guanidinated carbon dots (GCDs) and systematically characterized their immunomodulatory capabilities [192]. When applied to polyetheretherketone (PEEK) implants, GCDs significantly improved osseointegration in infected diabetic rats by combining antimicrobial effects with potent immune regulation.

Given that excessive ROS elimination may unintentionally suppress the signaling required for M2 macrophage induction, precise modulation of ROS levels has become an important design principle. To address this challenge, Wang *et al.* [193] engineered ultrathin tungsten oxide (WO_x_) nanoribbons with moderate ROS-scavenging ability. This fine-tuned nanozyme achieved an induction efficiency of 86.3% for M2 polarization of bone marrow-derived macrophages, outperforming previous nanozyme systems (Figure 10B). These findings underscore the need for balanced redox regulation to maximize immunotherapeutic outcomes in diabetic bone regeneration.

### Nanozyme for reducing local glycosylation

In addition to excessive ROS, the pathological accumulation of glucose in diabetic bone defects further amplifies inflammatory responses by driving nonenzymatic glycation and metabolic dysregulation. To mitigate the hyperglycemic burden, glucose oxidase (GOx) has been incorporated into biomaterial systems as a biological catalyst capable of oxidizing glucose into gluconic acid and hydrogen peroxide under high-glucose conditions. While this reaction effectively lowers local glucose levels, it simultaneously generates H_2_O_2_, creating a paradoxical risk of exacerbating oxidative stress. For this reason, contemporary nanozyme-based strategies frequently combine GOx with antioxidant components to achieve coordinated glucose depletion and ROS neutralization, thereby restoring a balanced redox state.

**Figure 11:**
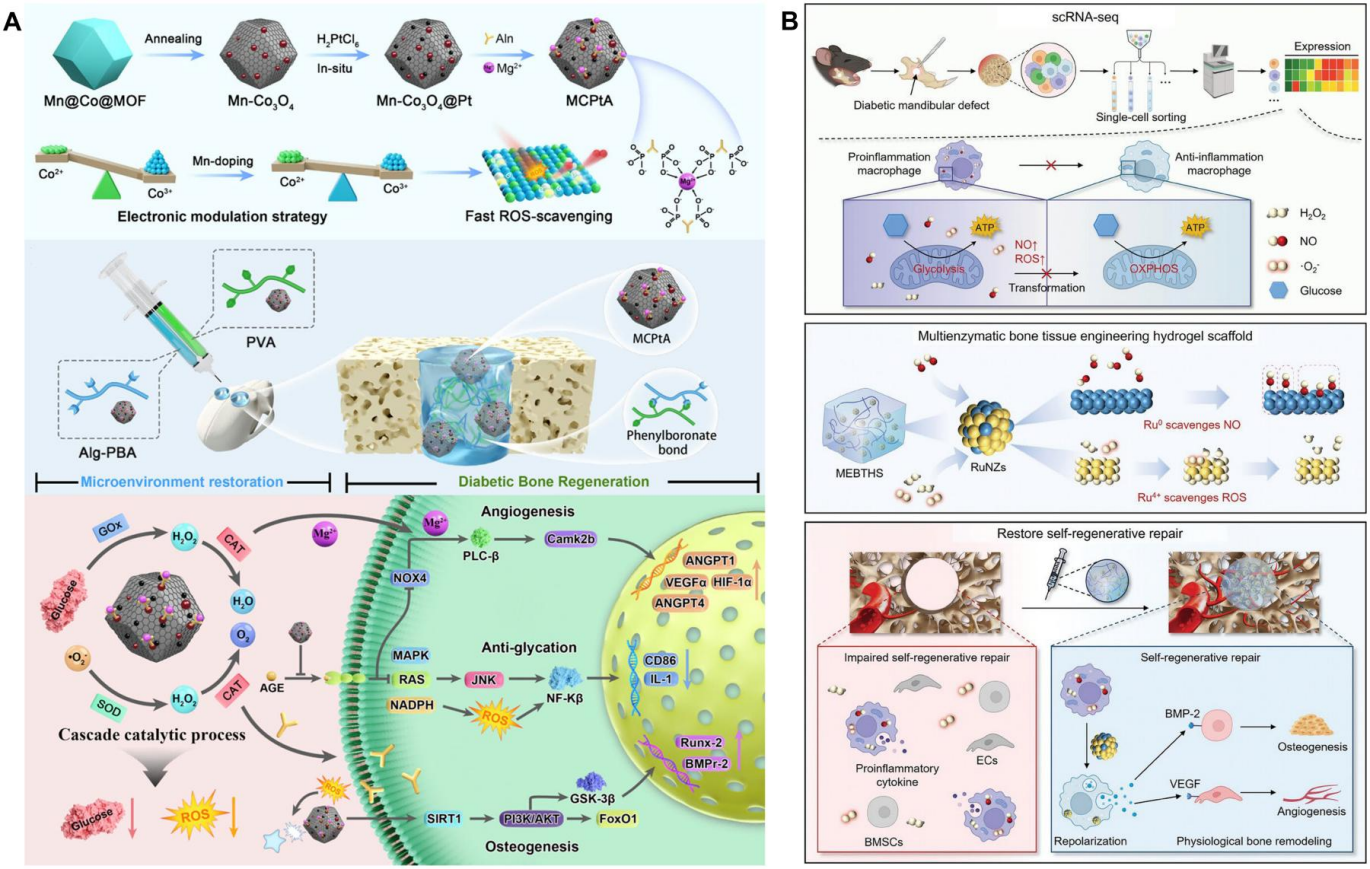
Multienzymatic nanozymes based strategy for diabetic bone regeneration. (**A**) Nanozyme Mn@Co_3_O_4_@Pt exhibits GOx-/SOD-/CAT-like catalytic activities, which achieves local glucose consumption, ROS scavenging and inflammation reduction. Reproduced with permission from Ref. [194]. Copyright 2025, American Chemical Society. (**B**) RuNZs exhibits SOD-/CAT-like and NO adsorption capacity, which suppress ROS-driven pro-inflammatory polarization and correct NO-mediated mitochondrial metabolic disruption. Reproduced with permission from Ref. [198]. Copyright 2024, Wiley.

Liu *et al*. [194] developed a bimetallic MOF-derived nanozyme (Mn@Co_3_O_4_@Pt) loaded with alendronate and Mg^2+^ ions, termed MCPtA, that exhibits GOx-, SOD- and CAT-like catalytic functions (Figure 11A). By simultaneously consuming glucose and scavenging ROS, MCPtA effectively reduced local glycosylation, promoted M2 macrophage polarization and achieved significant repair of diabetic bone defects *in vivo*. Similarly, Hui *et al.* [195] constructed a bimetallic nanozyme by integrating gold nanoparticles with intrinsic GOx activity and platinum nanoparticles with CAT activity, creating Au@Pt nanoparticles with dual enzymatic functions. This composite not only normalized the inflammatory microenvironment but also enhanced paracrine signaling in BMSCs under hyperglycemic conditions, particularly by upregulating TGF-β secretion.

Further advancements have led to the development of multifunctional nano-reactor systems. Zhang *et al*. [196] engineered a complex platform in which gold nanorods conjugated with β-cyclodextrin (β-CD) modified with methoxy polyethylene glycol and lipoic acid formed a host–guest complex (Au@CD) capable of encapsulating GOx via interactions with linear ferrocene derivatives. Through the synergistic interplay of NIR-triggered hyperthermia, controlled glucose oxidation and antioxidant effects, this system rapidly alleviated hypoxia while preventing pathological H_2_O_2_ accumulation. In another study, Yang *et al.* [197] designed CAT-assisted biomineralized calcium phosphate nanosheets (CaP@CAT NSs) to selectively decompose excessive H_2_O_2_. The nanosheets not only reduced pro-inflammatory cytokine expression in macrophages but also promoted osteogenesis through the release of Ca^2+^ and ${\text{PO}}_{4}^{3-}$ ions during scaffold degradation.

Collectively, these nanozyme-based designs highlight the therapeutic potential of synchronized glucose regulation and ROS scavenging to counteract glycation-associated inflammatory activation in diabetic bone repair.

### Nanozyme-based modulation of nitric oxide signaling

NO is an essential physiological regulator of mitochondrial bioenergetics and plays a critical role in shaping inflammatory responses, although its precise mechanistic pathways remain incompletely understood [201]. To delineate the metabolic complexity of macrophages in diabetic bone defects, Lin *et al*. [198] applied single-cell RNA sequencing and identified that excessive NO production prevents the metabolic transition from glycolysis to oxidative phosphorylation (OXPHOS), thereby inhibiting macrophage repolarization from a pro-inflammatory toward an anti-inflammatory phenotype (Figure 11B). These findings highlight the importance of NO homeostasis in restoring immune balance within diabetic microenvironments. Guided by these insights, Lin *et al*. developed ruthenium-based multienzymatic nanozymes (RuNZs) capable of simultaneously scavenging NO and ROS [202]. Ru^4+^ ions conferred robust CAT- and SOD-like activities, while Ru^0^ displayed strong NO adsorption capacity. These nanozymes were incorporated into a GelMA-based multienzymatic bone tissue engineering hydrogel scaffold (MEBTHS), enabling dual regulation of inflammatory pathways. By simultaneously suppressing ROS-driven pro-inflammatory polarization and correcting NO-mediated mitochondrial metabolic disruption, MEBTHS successfully induced macrophage phenotype switching through metabolic reprogramming. This dual-pathway immunomodulation restored bone repair in diabetic mandibular defect models and demonstrated a promising strategy for targeting metabolic inflammation in diabetic bone repair.

With advances in materials science, nanozyme-based platforms now exhibit multifunctional capabilities far beyond metabolic clearance alone. Through rational design, nanozymes can be endowed with osteoinductive and angiogenic activities, as well as antimicrobial functions mediated by photothermal or photodynamic mechanisms. These additional properties further optimize the microenvironment for bone repair under diabetic conditions. Future directions should emphasize leveraging synergistic combinations between nanozymes and conventional bone graft materials, as well as validating therapeutic efficacy in large-animal models to accelerate clinical translation.

## Immune-instructive physicochemical materials mimicking endogenous signaling

Endogenous biophysical and biochemical signals, including electrical cues, mechanical stimuli and gaseous mediators, undergo substantial fluctuations during tissue injury and repair. These signals profoundly influence immune regulation and bone regeneration by shaping microenvironmental conditions and triggering intracellular biochemical pathways [203]. Physical stimulation, in particular, plays a central role in skeletal homeostasis, as mechanical or electrical cues are converted into intracellular signals that dynamically coordinate osteogenesis and osteoclastogenesis. A classic example is the influence of implant surface properties such as roughness, wettability and surface charge on osseointegration. Specifically engineered surfaces like TiO_2_ nanotubes (TNT) can modulate macrophage responses to pro-inflammatory stimuli and direct them toward an anti-inflammatory phenotype. Research has shown that TNT surface can activate forkhead box O1 (FOXO1) to mitigate oxidative stress, and induce antioxidant activities and anti-inflammatory response pathways, thereby promoting macrophage polarization toward an anti-inflammatory phenotype and enhancing osteogenesis through macrophage-MSC crosstalk in the diabetic microenvironment [204, 205]. However, diabetic microenvironments characterized by hyperglycemia, oxidative stress and chronic inflammation demand more robust immunoregulatory interventions. Numerous attempts have been made to address this unmet need by mimicking endogenous biophysical and biochemical signals, aiming to reprogram the dysregulated diabetic microenvironment. Representative strategies and their therapeutic outcomes are outlined in Table 4.

**Table 4:** Mimicking endogenous signals to affect the healing process for diabetic bone regeneration.

Endogenous signals	Biomaterials	Immune cell regulation mechanism	Ref.
Mechanical signals	TiO_2_ nanotube coating on Ti implants	FOXO1-induced oxidation resistance and anti-inflammatory osteoimmunity	[204]
Bioelectrical signals	Ferroelectric BaTiO_3_/P(VDF-TrFE) nanocomposite membrane	Biomimetic electrical microenvironment that can switch macrophage phenotype from M1 into M2 by suppressing expression of AKT2 and IRF5 within the PI3K-AKT signaling pathway	[206]
	PCL and KNN electroactive membrane	Establishing temporal immunomodulation strategy accordingly to promote stem cell recruitment by moderate M1 macrophages at early stage as well as osteogenic differentiation by abundant M2 macrophages at late stage	[207]
	PDA decorated polarized graphene oxide/P(VDF-TrFE) ferroelectric membrane	Boosting ROS elimination and reversing inflammatory status, contributing to strengthened osteogenic differentiation of MSCs and M2 polarization of macrophages	[208]
Gaseous signals	Poly(3,4-ethylenedioxythiopene)-assembled polydopamine mediated silk microfiber network and a hydrogen sulfide sustained-release system utilizing bovine serum albumin nanoparticles	The synergistic effects of hydrogen sulfide gaseous-bioelectric coupling promote bone formation by amplifying autophagy in periodontal ligament stem cells and modulating macrophage polarization via lipid metabolism regulation	[209]
	H_2_S-delivery hydrogel	Glucose-responsive H_2_S release promoting mitophagy and preventing macrophage senescence progression	[209]

Recent advances in bioelectronic and electroactive biomaterials have introduced powerful strategies that leverage microenvironmental electrical interactions and intracellular signaling modulation to restore immune homeostasis in diabetes [210].

In 2016, Zhang *et al.* [211] developed a polarized ferroelectric nanocomposite membrane based on corona-treated barium titanate (BaTiO_3_) nanoparticles, which endowed the membrane with a negatively charged inner surface. This structure created a stable localized electrical microenvironment when interfaced with the negatively charged bone wall. Follow-up studies demonstrated that this bioinspired electrical milieu effectively suppressed protein kinase B (AKT2)-interferon regulatory factor 5 (IRF5) signaling in macrophages under diabetic conditions, thereby inducing anti-inflammatory polarization and enhancing bone repair [206].

**Figure 12:**
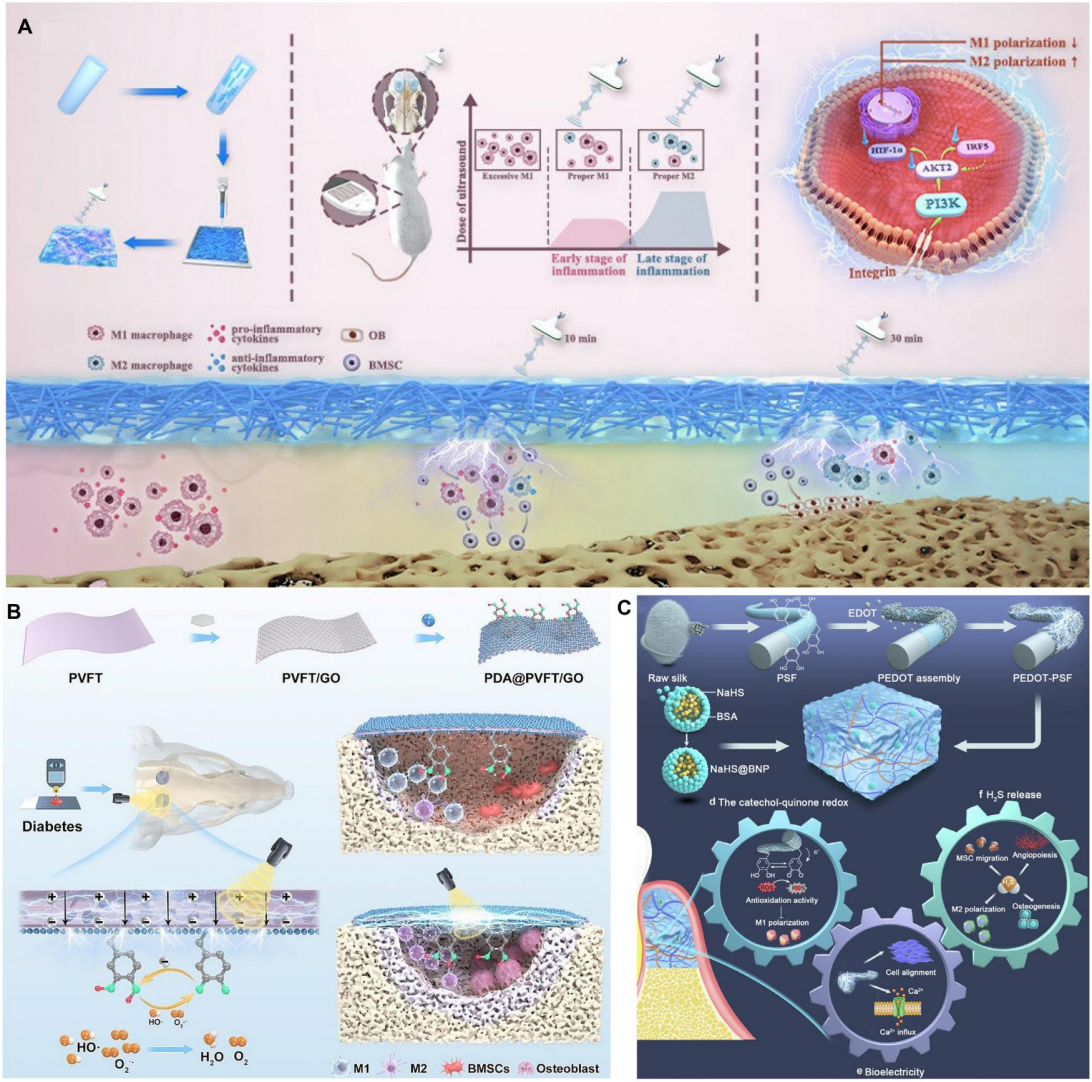
Endogenous signaling mimicking for diabetic bone regeneration. (**A**) Ultrasound-responsive electroactive membrane generated controlled electrical signals that dynamically reprogrammed macrophage phenotypes via the AKT2-IRF5/HIF-1α axis reproduced with permission from Ref. [207]. Copyright 2023, American Chemical Society. (**B**) Piezocatalytic P(VDF-TrFE) membrane regenerated its antioxidant capacity under ultrasound stimulation through charge polarization effects. Reproduced with permission from Ref. [208]. Copyright 2024, Elsevier. (**C**) PEDOT fibers encapsulated NaHS, enabling dynamic redox cycling through electron transfer to promote intracellular antioxidant defenses and inflammation control. Reproduced with permission from Ref. [209]. Copyright 2024, Springer Nature.

Building on this concept, the same groups engineered an ultrasound-responsive electroactive membrane composed of electrospun polycaprolactone (PCL) and potassium-sodium niobate (KNN). Upon ultrasound stimulation, the membrane generated controlled electrical signals that dynamically reprogrammed macrophage phenotypes via the AKT2-IRF5/HIF-1α axis, ultimately rescuing inflammation-impaired osteogenesis in diabetic settings (Figure 12A) [207]. They also created a piezocatalytic composite by integrating polarized graphene oxide and polydopamine onto a poly (vinylidene fluoridetrifluoroethylene) [P(VDF-TrFE)] substrate. Polydopamine neutralized ROS through chemical scavenging, while the piezoelectric matrix regenerated its antioxidant capacity under ultrasound stimulation through charge polarization effects (Figure 12B) [208]. This platform provided sustained ROS regulation and effectively reconstructed a regenerative immune microenvironment in diabetic bone defects.

Other research has explored smart redox-regulating and gas-releasing systems inspired by endogenous gaseous mediators. Fang *et al.* [209] engineered conductive poly(3,4-ethylenedioxythiopene) (PEDOT) fibers modified with polydopamine, enabling dynamic redox cycling through electron transfer to promote intracellular antioxidant defenses (Figure 12C). To complement this effect, they encapsulated sodium hydrosulfide (NaHS) within albumin nanoparticles to achieve controlled release of H_2_S, an endogenous gaseous signaling molecule involved in inflammation control, stem cell regulation and bone metabolism [212]. This dual-mechanism strategy effectively mitigated oxidative stress and restored immune balance in diabetic tissues. Expanding the therapeutic relevance of H_2_S, Zhang *et al*. [73] developed HydroWrap, a glucose-responsive H_2_S-delivery system capable of alleviating mitochondrial dysfunction and preventing macrophage senescence in T2DM. This intervention significantly accelerated femoral fracture healing and improved bone quality in diabetic mice.

Overall, biomaterials designed to mimic endogenous signaling represent a rapidly expanding class of intelligent immunomodulatory platforms. Their versatility allows real-time microenvironment monitoring, prevention of infection, enhancement of cell function and promotion of tissue regeneration. Their programmable responsiveness and multistimuli synergistic mechanisms are particularly advantageous for addressing the fluctuating and hostile diabetic milieu. Nonetheless, further refinements are needed to improve their stability, optimize their adaptability to complex diabetic conditions and elucidate their detailed immunoregulatory mechanisms.

## Conclusion and perspective

Immune dysregulation is now widely recognized as a central pathological mechanism underlying impaired bone regeneration in diabetes. Hyperglycemia-driven oxidative stress, advanced glycation reactions, sustained hypoxia, altered metabolic programming and paracrine dysfunction collectively disrupt osteoimmune homeostasis, resulting in prolonged inflammation, impaired angiogenesis and defective osteogenesis. Against this background, biomaterials specifically designed to modulate immune responses have emerged as a promising category of therapeutics for diabetic bone repair. These advanced platforms can reprogram macrophage phenotypes, promote the transition to anti-inflammatory and pro-regenerative microenvironments, eliminate excessive glucose and ROS, deliver immunoregulatory cytokines or bioactive molecules and mimic endogenous biophysical and biochemical signaling. By reshaping the inflammatory milieu, these materials restore angiogenic-osteogenic coupling and enhance the intrinsic regenerative capacity of diabetic bone tissue. Despite these encouraging advances, the translation of immunomodulatory biomaterials into clinical applications still faces many challenges.

### Standardized scalable fabrication

A central obstacle in this field is the lack of technologies for large-scale and uniform biomaterial production. Most biomaterials previously discussed rely on small-batch, manual laboratory fabrication, which is incompatible with industrial requirements for standardized, repeatable processes and cost efficiency. Advanced manufacturing techniques, such as microfluidics and 3D printing, have been increasingly applied to biomaterial manufacture to provide standardized production solutions. However, the large-scale production of cell derivatives and EVs without compromising their functionality remains an unresolved challenge. Conventional laboratory-scale isolation methods, such as ultracentrifugation and size-exclusion chromatography, suffer from significant yield variability and unstable concentrations of active components. Furthermore, the inherent heterogeneity of cell states (e.g. donor variability, passage differences) often results in functional inconsistencies across different EV batches [213]. Emerging platforms like bioreactor technologies and microfluidic systems represent viable solutions for industrializing clinical-grade EVs fabrication. Additionally, strategies such as genetic engineering, chemical modification and physical refinement offer promising avenues for optimizing EV quality control. These systems may enable standardized, repeatable processes that maintain uniform quality and bioactivity of EVs [214]. Furthermore, sterilization, storage and transportation methods capable of maintaining the structural and functional integrity of materials are crucial for realizing their therapeutic potential, particularly for cell derivatives and cytokines.

### Long-term safety and stability

The majority of reported systems have been evaluated only in short-term rodent models. These models exhibit rapid healing dynamics that only partially recapitulate the chronic and systemic features of human diabetes. Although biomaterials are generally designed using raw materials with low immunogenicity and cytotoxicity, significant barriers to clinical translation persist. These include the immunogenicity of xenogeneic hydrogel materials (potentially harboring endotoxins, protein contaminants or residual antigens) and metabolism and potential toxicity of nanozymes and metal-ion materials *in vivo*. These considerations are particularly critical in the diabetic population, who often present with systemic immune dysregulation and an increased susceptibility to infection. Long-term studies in large-animal models, particularly those mimicking T2DM with comorbidities, are essential to establish safety, pharmacodynamics, biodistribution and long-term immune outcomes. Furthermore, the stability of carrier materials across biological environments, alongside their biodegradability, are critical factors. Ideally, therapeutic scaffolds should exhibit degradation rates synchronized with the pace of tissue regeneration. This is especially pertinent in bone regeneration, where material residues can compromise the structural integrity and mechanical strength of the repaired tissue. To date, however, research focusing on this specific dimension remains sparse.

### Therapeutic precision

Therapeutic precision requires biomaterials to ensure that drugs or therapeutic effects act at the right time, in the right spatial location and at a precise dosage, while minimizing side effects on healthy tissues. Since nanozymes generally lack inherent catalytic specificity, targeting delivery and activation can avoid some unnecessary and even harmful catalytic reactions in organisms. Current strategies include employing host cell-targeted bioorthogonal nanozymes to facilitate cellular uptake and localized intracellular function. Given that disruptions to intracellular homeostasis are often localized within specific organelles, developing nanozymes capable of precise sub-cellular targeting may contribute to further enhancing therapeutic efficacy. Alternatively, designing responsive carriers allows nanozymes to be activated exclusively within microenvironments characterized by elevated ROS or glucose levels [215]. While EVs possess innate targeting capabilities due to their membrane-bound ligands and receptors, which makes them easily sequestered by macrophages, surface functionalization is typically required to enhance delivery specificity to other cell types, such as stem cells. For pharmaceuticals, bioactive factors, and metal ions, static release profiles fail to accommodate the dynamic transition of the immune response from an early pro-inflammatory phase to a late pro-repair phase during wound healing. To achieve temporal precision, hierarchical-release hydrogels responsive to endogenous ROS or glucose have been proposed. The aforementioned IL-10/BMP-2 sequential release system exemplifies a promising approach to temporal orchestration. Subsequent studies could explore other logical pairings, such as SDF-1-mediated recruitment followed by VEGF-driven angiogenesis, to synchronize the recovery of the diabetic osteoimmune microenvironment. Meanwhile, the precise spatial-temporal and dosage-controlled regulation of these systems remains a significant challenge that warrants further investigation. The wound microenvironment in diabetic patients is characterized by both inter-individual variability and temporal shifts across different stages of progression. This complexity necessitates the establishment of robust clinical monitoring metrics to guide therapeutic applications and enable the selection of appropriate stimuli-responsive dressings tailored to the specific status of the wound.

### Systematic assessment

The lack of standardized evaluation criteria complicates the comparison of analogous materials. Differences in animal models, defect types, dosing strategies, readout timepoints and immune assays make it difficult to determine which platforms possess the most robust translational potential. Establishing harmonized guidelines for immunomodulatory biomaterials, similar to standards used in bone graft or implant evaluation, will be crucial for the systematic assessment of efficacy and mechanism.

### Future perspective

Looking ahead, the successful translation of immunomodulatory biomaterials will rely heavily on deep interdisciplinary collaboration between materials scientists, clinicians, immunologists and bioengineers. A mechanistic understanding of diabetes-associated immune alterations must guide the rational identification of therapeutic targets. High-resolution technologies such as single-cell omics, spatial transcriptomics and metabolic profiling will continue to clarify the cellular and molecular determinants of impaired diabetic bone repair, enabling the design of biomaterials with precise immune-instructive properties. Growing evidence highlights the critical contribution of the neuro-immune axis to diabetic immunopathology. Consequently, developing advanced materials to modulate this axis (e.g. through CGRP-mediated signaling) represents a potent strategy for restoring immune homeostasis and promoting bone repair. While current biomaterial-based strategies predominantly target the downstream pathological sequelae of hyperglycemia, the underlying epigenetic modifications of gene expression remains largely unaddressed. Investigating the epigenetic landscape of diabetic bone repair is essential for deciphering the mechanisms of ‘trained immunity’ and identifying untapped therapeutic avenues. For biomaterials that currently exhibit significant therapeutic potential, such as nanozymes and EVs, developing scalable manufacturing technologies and conducting rigorous, long-term biosafety assessments will be essential to bridging the gap from bench to bedside. To achieve precision delivery that synchronizes with the distinct phases of bone regeneration, future therapeutic strategies must adopt multifactor integrated, logic-gated designs. By ensuring spatiotemporal precision in drug release, these systems can optimize therapeutic efficacy while minimizing potential toxic effects. Building upon these foundations, the synergistic orchestration of diverse biomaterial modalities, such as the integration of biomimetic signaling, sustained drug delivery and nanozyme-mediated catalysis into a single platform, may enable a holistic remodeling of the diabetic pro-inflammatory microenvironment. Moreover, integrating material-based approaches with systemic diabetes management, including glycemic control, metabolic modulation and anti-inflammatory therapies, may yield synergistic outcomes that surpass the benefits of either strategy alone.

In summary, immunomodulatory biomaterials offer a transformative paradigm for treating diabetic bone defects. By restoring osteoimmune homeostasis and reactivating endogenous repair pathways, these materials hold the potential to substantially improve therapeutic outcomes and quality of life for patients with diabetes. Continued advances in mechanistic understanding, material design and translational research will ultimately pave the way for their successful clinical implementation.
